# Unveiling the Challenge of Evaporator Design in Clean Water Production Promoted by Superabsorbent Hydrogels and Sunlight

**DOI:** 10.1021/acsami.5c20819

**Published:** 2026-01-05

**Authors:** Umamah Amir, Sonia Lanzalaco, Kathrin Harre, Alba Àgueda, Maria M. Pérez-Madrigal, Ignasi Sirés, Elaine Armelin

**Affiliations:** † IMEM-BRT Group, Departament d’Enginyeria Química, EEBE, 16767 Universitat Politècnica de Catalunya (UPC), C/Eduard Maristany, 10-14, Ed. I, Second Floor, Barcelona 08019, Spain; ‡ Barcelona Research Center in Multiscale Science and Engineering (CCEM), EEBE, 16767 Universitat Politècnica de Catalunya (UPC), C/Eduard Maristany, 10-14, Basement S-1, Barcelona 08019, Spain; § Polymer Chemistry Laboratory, Faculty of Agriculture and Environment Chemistry, Hochschule Für Technik und Wirtschaft Dresden (HTWD), Friedrich-List-Platz 1, Dresden D-01069, Germany; ∥ CERTEC Group, Departament d’Enginyeria Química, EEBE, Universitat Politècnica de Catalunya (UPC), C/Eduard Maristany, 10-14, Ed. I, Fifth Floor, Barcelona 08019, Spain; ⊥ Laboratori d’Electroquímica dels Materials I del Medi Ambient, Departament de Ciència de Materials I Química Física, Secció de Química Física, Facultat de Química, 16724 Universitat de Barcelona, Martí I Franquès 1-11, Barcelona 08028, Spain

**Keywords:** thermosensitive hydrogels, conducting polymers, advanced materials, sustainable energy, desalination

## Abstract

Climate change is affecting water availability and the supply. This situation is particularly worrying in Mediterranean area countries, where droughts are becoming increasingly long and severe. Herein, a superabsorbent porous hydrogel composed of thermoresponsive hydrogel (TSH) poly­(*N*-isopropylacrylamide) (PNIPAAm), copolymerized with poly­(acrylamide) (PAAm) and modified with poly­(3,4-ethylenedioxythiophene) polystyrenesulfonate (PEDOT/PSS), as a solar absorber, is presented. This superabsorbent hydrogel optimizes water uptake and provides long life stability through a continuous supply of water to the evaporation surface, promoted by its thermosensitivity property and light absorption efficiency with a very low amount of photothermal material (1 wt %). The fine-tuning of both the hydrogel composition and the solar vapor generator (SVG), assisted by a metallic reflector, results in an impressive evaporation rate (ER) of 6.34 kg·m^–2^·h^–1^. This configuration minimizes the heat losses and allows maintaining the ER high, if compared to other SVG architectures. The hydrogel also exhibits strong removal capacity for monovalent cations and transition metals as well as reusability properties under stable multiple evaporation-swelling cycles, thanks to its good covalent interpenetrating network and its mechanical integrity. This superlative performance significantly expands the potential applications of porous hydrogels in clean water production, which are moved by sunlight irradiation and seawater, two abundant natural resources.

## Graphical abstract

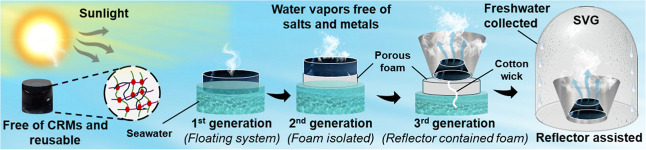

## 1 Introduction

The efficacy of porous materials to convert seawater into freshwater by applying solar-driven desalination (SDD) has been explored in several works^1–5^ and has been consolidated as a green alternative when compared to other desalination methods.^6^ SDD follows the natural water cycle; when the porous material impregnated with brine solutions is heated by solar radiation, a cloud is formed, which subsequently condenses and precipitates. The overall result of the process is desalination of the water. Over the past decade, researchers have centered in achieving the best material, with the lowest water vaporization energy^7,8^ and the highest evaporation rate (ER) per unit of area and hour, under 1 sun (sunlight power of 1 kW/m^2^). However, only few works have reported the real amount of drinkable water recovered after SDD.^9^ For instance, Qu and Yu, among the pioneers in the application of hydrogel materials for SDD^10,11^ proposed, for the first time, an efficient prototype for outdoor installations, able to work continuously and under real solar radiation.^12^ The membrane they used was composed by poly­(vinyl alcohol) (PVA), a superabsorbent gel, modified with poly­(pyrrole), the photothermal absorber component, and the efficiency of freshwater production was estimated in 18–23 L·m^–2^·day^–1^ (i.e., 0.75–1.0 L·m^–2^·h^–1^). In another study, a condensation chamber was used to collect the generated freshwater with a 3D-coiled structure fabricated with poly­(pyrrole)-coated PVDF membrane.^13^ However, although the process is highly sustainable, it is still far from achieving the ideal volume of output water required to scale-up such solar evaporators.

So far, we have learned important lessons: (i) hydrophilic hydrogels, which have interconnected porous structures and a great capacity for water uptake, are good candidates;^11,14–20^ (ii) the presence of photothermal absorber materials, as for example, carbon-based compounds (carbon black, graphene, carbon nanotubes, among others)^21–23^ conducting polymers (CPs: polypyrrole, polyaniline, and poly­(3,4-ethylenedioxythiophene))^24–26^ and UV-absorber molecules (tannic acid, chromogenic agents, and metal oxides)^27–29^ is mandatory; (iii) the solar power is obviously an unambiguous condition, and high power intensity usually enhances the ER; and, most importantly, (iv) the solar vapor generator (SVG) design is decisive for a good SDD performance. So far, a great number of reviews in SDD insists in comparing the energy conversion efficiencies of different material architectures (1D, 2D, and 3D water paths) assembled in evaporators with completely dissimilar dimensions.^8,30^ Even though, there is a big controversy regarding the methodologies used to calculate the thermal efficiencies (usually denoted as “eta”, η).^31,32^ Some researchers have proved that η higher than the theoretical limit (100%) is possible because the system is not at constant temperature and pressure and, therefore, does not follow the thermodynamic laws. Nevertheless, the high energy losses (radiation, reflection, conduction, or diffusion) hinder the prototypes from achieving such values. By the contrary, the reason for having η > 100% is intrinsically related to the surface area (known as projected area, *A*_proj_), considered in the ER calculations and, consequently, in the η data. Thus, it is obvious that either ER or η can only be used to contrast materials with similar geometry and the same SVG assembly.

To date, there is a consensus that 3D evaporators have better energy efficiencies than 2D and 1D evaporators. Nowadays different strategies are being introduced to maximize the water collection, and the tendency is to move to a dual SDD technologies, with the combination of photo- and electrothermal evaporation systems.^33,34^ For instance, recently, Liu and coauthors reported a powerful improvement for the water solar cell evaporator by coupling a “reflector-assisted tool”, fabricated in aluminum foil and with an improved wall inclination of 45°, to enhance the steam production.^35^ This work marked an inflection point in SDD technology because it proved that high temperatures can favor much more the flux of vapor rather than evaporators based on temperature gradients, usually from the bottom (cold) to top (hot) surfaces. They also demonstrated that compounds with higher hydrophilicity present higher mass losses. Moreover, the incorporation of a “reflector” accessory optimizes the material energy losses and maximizes the ER when compared to the classical SVGs, composed by a material: (i) in direct contact with water (so-called “floating mode”) and (ii) located on the top of an insulator foam floating inside a beaker with water (called “self-contained” system). In our past studies, we adopted a nonfloating mode, with poly­(*N*-isopropylacrylamide) (PNIPAAm) copolymer hydrogel in the bottom of the vessel, without direct contact with seawater, unless it was absorbed before starting the SDD experiments. This configuration also allowed reaching very high evaporation rates thanks to the thermoresponsiveness and shrinkable property of NIPAAm units.^25,36^ Therefore, we hypothesized that the performance of our copolymer hydrogel in SDD could be highly enhanced if configured following the new design using metallic “reflector” tools (i.e., the third generation of SVGs).^35^

Herein, we report the preparation of superabsorbent hydrogels, with different compositions of NIPAAm monomer and a highly hydrophilic counterpart, acrylamide (AAm), able to establish more hydrogen bond interactions with water clusters, and their implementation in four models of SVG devices. The synergy between the novel materials and the superevaporator reflector-assisted system was able to separate the salts and impurities present in seawater, as well as recovering an important volume of freshwater per hour. Indeed, we employed a similar SVG system as that reported by Liu and co-workers, using an innovative thermoresponsive hydrogel (TSH) able to simultaneously superabsorb water and achieve huge amounts of vapor, if compared to similar hydrogels with different contents of TSH. Furthermore, the beneficial effect brought by the metallic reflector has been demonstrated. Its implementation allowed us to compare the role of this new SVG in thermal conduction enhancement across the material (by means of thermocouple probes).

Overall, although the volume of freshwater obtained cannot compete yet with traditional desalination methods, this work serves as an inspiration for the scientific community, highlighting the importance of standardizing the water solar cell configuration to better discriminate solar efficiencies and ERs among different materials.

## 2 Experimental Section

### 2.1 Materials

*N*-Isopropylacrylamide (NIPAAm) monomer (purity 99%, CAS 2210-25-5), acrylamide­(AAm) monomer (purity 99%, CAS 79-06-1), poly­(3,4-ethylenedioxythiophene)-poly­(styrenesulfonate) (PEDOT/PSS) conductive grade polymer (1.3 wt % dispersion in H_2_O, MFCD07371079), N,N,N′,N′-tetramethylethylenediamine (TEMED) initiator (Reagent Plus 99%, CAS110-18-9)), and *N*,*N*′-methylenebis­(acrylamide) (MBA) cross-linker (Reagent Plus 99%, CAS 110-26-9) were supplied by Sigma-Aldrich (Spain). Ammonium persulfate (APS) catalyst (purity 98%, CAS 7727-54-0) was provided by Honeywell Fluka, and Milli-Q water grade (0.055 S cm^–1^) was used in all synthetic processes. N_2_ gas was used for the radical polymerization reactions and was of pure grade (99.995% purity).

### 2.2 Synthesis of PNIPAAm-PAAm and PNIPAAm-PAAm/PEDOT/PSS Hydrogels

By modifying a previously published synthesis,^37^ six different hydrogel materials were prepared using specific chemical compositions and concentrations. NIPAAm was used at 250, 500, and 600 mM, while AAm was added at 500, 250, and 150 mM, thus giving three different molar ratios of 1:2, 2:1, and 4:1, respectively, for PNIPAAm/PAAm compositions. Control samples do not have a conducting polymer. For samples containing PEDOT/PSS, 1.2 mL (1 wt %) was added in the reactor to obtain the photothermal property required for SDD tasks. The cross-linker MBA was kept at 0.53 mM, TEMED at 2.77 mM, and APS at 2.77 mM (from stock solution of 370 mM). The solvent used was Milli-Q water, 19.5 mL for the non-PEDOT hydrogels, and 18.3 mL for the PEDOT containing hydrogels.

In the initial step, NIPAAm, AAm, MBA, PEDOT/PSS (if applicable), TEMED, and Milli-Q water were mixed for 30 min inside the closed reactor with a magnetic stirrer. PEDOT/PSS was previously sonicated for 20 min in order to obtain a better dispersion. The closed reactor was then sealed and bubbled with pure nitrogen gas for 30 min to obtain the inert atmosphere. Then the reactor was placed in the ice bath for 20 min to lower the temperature, and the initiator (APS) was injected using a syringe fitted with a needle to start the radical reaction. The solution was stirred to ensure the proper mixing of APS and then extracted using a syringe and placed into the molds. Following a 1 h reaction at 30 °C, the resultant samples were demolded and purified onto 400 mL of Milli-Q water washed for 24 h for purification. The chemical structure of all the components and photographs of the hydrogels with cylindrical shapes are shown in Scheme 1a,b.

**Scheme 1:**
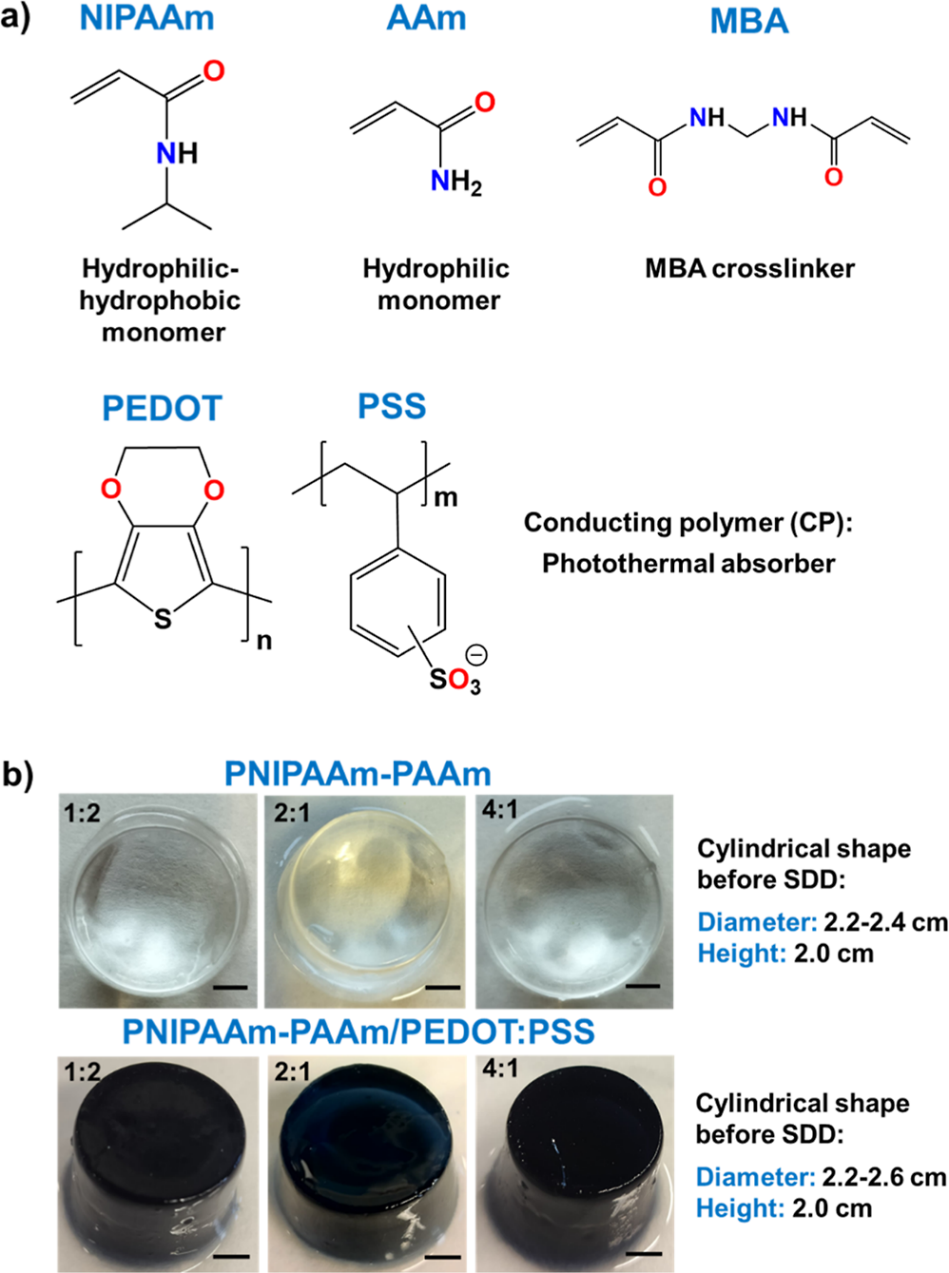
(a) Chemical Structures of *co*-Monomers (NIPAAm and AAm), Cross-Linker (MBA), and Photothermal Absorber (PEDOT/PSS). (b) Photographs of SAHs Freshly Synthesized. Top Row: PNIPAAm-PAAm Hydrogels and Bottom Row: PNIPAAm-PAAm/PEDOT/PSS Dark Hydrogels for SDD Experiments. The Compositions of the Hydrogels and the Scale Bars (0.5 cm) Are Indicated in the Images

### 2.3 Physicochemical Characterization

Several characterization methodologies were employed to confirm the swelling and physical-chemistry properties of PNIPAAm-PAAm and PNIPAAm-PAAm/PEDOT/PSS hydrogels. Detailed experimental procedures are included in the Supporting Information.

### 2.4 Solar-Driven Evaporation Study in Open Air Devices

To conduct the solar evaporation study, first synthetic seawater was prepared. The seawater is a standard solution (“Sea-Salt” ASTM D1141-98) containing the following salts: sodium bicarbonate (0.477%), potassium bromide (0.238%), sodium chloride (58.490%), magnesium chloride (26.460%), potassium chloride (1.645%), calcium chloride (2.765%), and sodium sulfate (9.750%), supplied by Panreac S.A. Those compounds were mixed in milli-Q water and stirred until fully dissolved to obtain 1L of solution. The hydrogels were submerged in seawater for 2 h for salt water swelling before the SDD experiments, which represents the time for stable water absorption (constant weight). Afterward, they were subjected to solar-driven evaporation in four different SVG arrangements, according to Scheme 2 illustration.

**Scheme 2:**
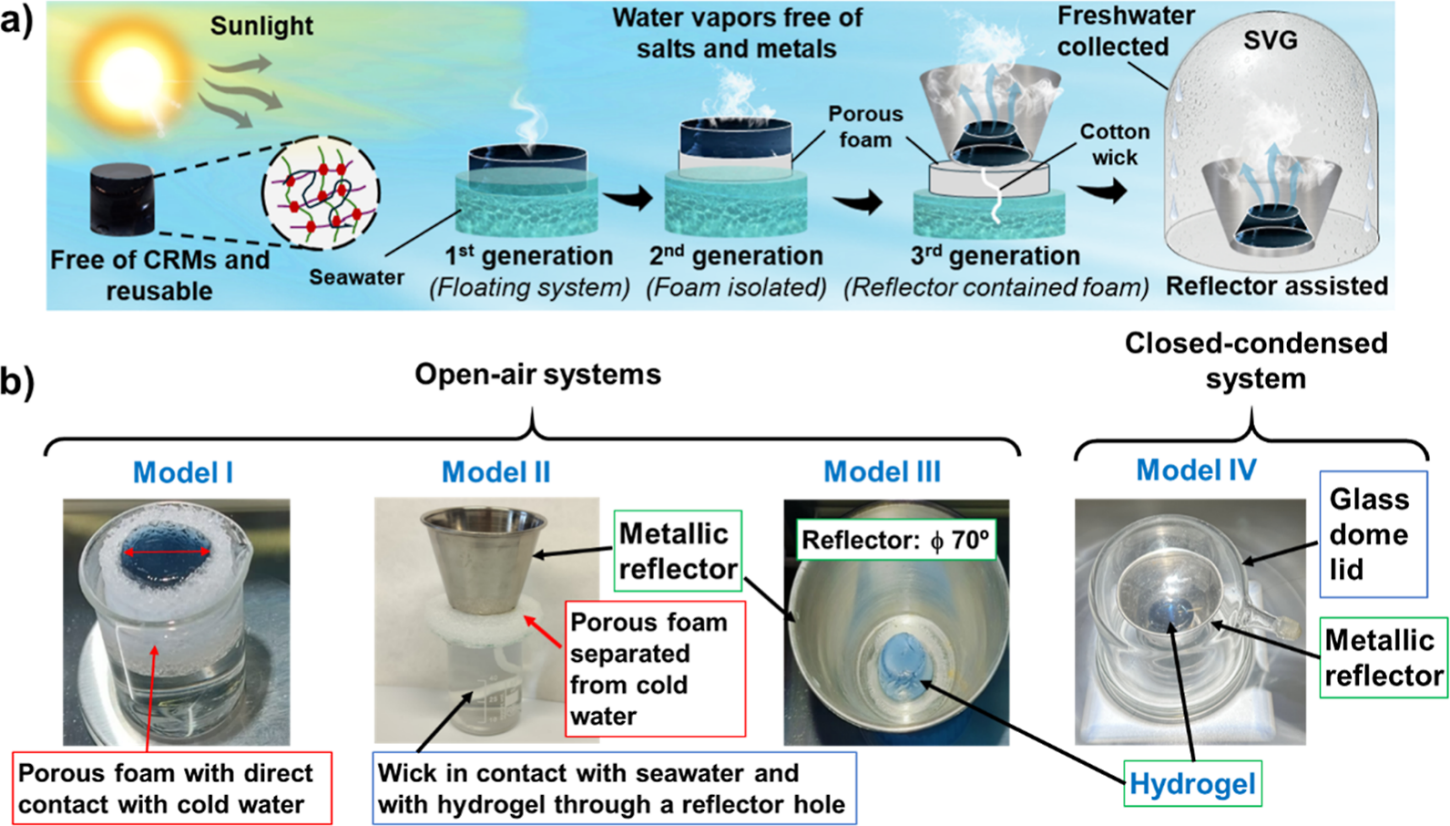
(a) Schematic Illustration of the Generational Evolution of SDD (SDD) Systems, with Reflector-Assisted as 3rd Generation and the Most Promising to Rationally Minimize Heating Losses in SVG. The Material Hydrogels Developed Here Were Represented in Scheme 1 and Are Free of Critical Raw Materials (CRMs). (b) Photographs of the Three Different SVG Cell Arrangements Composed by Open-Air Models: Model I: “Self-Contained” in a Porous Foam; Model II: “Reflector-Contained” with Insulating Foam as Separator Element of Reflector Recipient with Cold water, and a Cotton Wick for the Continuous Seawater Flow Up Supply; Model III: “Reflector-Assisted”, without Contact of the SAH with the Cold Water (Fixed Volume of Seawater Swollen by the TSH); and Model IV, a SVG Arrangement Composed by “Reflector-Assisted” Tool in a Closed-Condensation System

#### 2.4.1 **Model I** (“Self-Contained” Foam)

The sample holder is constructed with polystyrene (PS) open-cell foam (recycled material used in packaging) and was cut to the dimensions of the hydrogel cylinders employed in the experiment, such as the photograph shown in Scheme 2b. The hydrogels are sustained by the same plastic foam on the bottom of the holder. Although we employed an open-cell foam (macroporous foam), it was perforated with a series of punctures to facilitate the flow of water into the hydrogel, from bottom to top. The sample holder, with the cylindrical hydrogel inside, was positioned within a small beaker, which was precisely fitted to the beaker filled with cold seawater (type of SDD process: continuous supply of water).

#### 2.4.2 **Model II** (“Reflector-Contained” Foam)

In this SVG construction, the hydrogel was placed in a metallic reflector element, and it was positioned in a beaker containing seawater (Scheme 2b). PS plastic foam was used to separate the reflector reservoir from the seawater positioned at the bottom of the beaker, to avoid contact with cold water, and to minimize thermal conduction losses. It also provided structural stability for the overall system. A cotton wick passed through the open-cell foam and the reflector and touched the bottom of the hydrogel, connecting the hydrogel to water and allowing water to move upward from the beaker through capillary action (type of SDD process: continuous water supply). The reflector was made of stainless steel 316L and had the following dimensions: a bottom diameter of 4 cm (solid, with a 3 mm hole for wick assembly), a top diameter of 8 cm (open), a height of 5 cm, and an inclination angle of 70°.

#### 2.4.3 **Model III** (“Reflector-Assisted” Device)

In this configuration, the hydrogel was directly placed on the metallic reflector without the supply of continuous water (type of SDD process: batch, water is supplied only by swelling, before SDD) (Scheme 2b). Therefore, there was no contact with cold seawater. The reflector was equal to that reported in Model II, without the bottom hole. Cylindrical hydrogels were left for 2 h in seawater for swelling before any experiment in order to substitute any residual pure water and fill them with the desired solution. It represents the third generation of SVGs (Scheme 2a).

All of those configurations or models were positioned on an electronic analytical weighing balance (Entris II Sartorius balance, model BCE224i-1S, precision: 0.1 mg) to monitor the mass change during the evaporation process in the SDD experiment. Moreover, the samples were subjected to a 2 h preswelling condition, in 50 mL of seawater solution and then moved to the sample holders explained for each SVG model.

A SunLiteTM sun simulator (ABET technologies) with a 100 W xenon arc lamp was used to perform the sun simulation. A Si reference cell was used to calibrate the simulator height in advance to guarantee a 1 sunlight intensity (1 kW/m^2^) in the hydrogel top surface. Also, a thermographic camera (Optris PI640) was used to monitor temperature differences of the hydrogel during the irradiation period. The camera was focused on the top surface of the hydrogel and was able to measure the temperature of the hydrogel surface in real time through a connection with a computer.

During the 4 h irradiation, the thermographic camera captured an image of the heat distribution every 5 min, and the mass loss was measured every 20 min. Prior to and following each radiation interval, the diameter of the hydrogel was measured due to the shrinkage of PNIPAAm-PAAm counterparts under heating conditions. The irradiated hydrogels were recovered in deionized water over a 24 h period.

Using all 3 models, temperature measurements were carried out with a data acquisition device (USB-2408-2AO, Measurement Computing). Two type K thermocouples (1 mm diameter) were employed to monitor the temperatures at the top and bottom surfaces of the hydrogel during a 4 h solar irradiation experiment involving the PEDOT/PNIPAAm/PAAm (4:1) hydrogel. Data logging and real-time visualization were conducted by using DAQami software to record the temperature profiles throughout the irradiation period.

The **ER_2D_** was determined by quantifying the mass of water evaporated divided by the surface area (***A***_**surface**_) exposed to direct sunlight (the projected area of the upper surface in m^2^ is used, and the hydrogel shrinkage diameter was considered), per unit of time (***t***, in hour), as indicated in eq 1.^35^

1 $${ER}_{2D}=\frac{\Delta m_{sample}}{A_{surface}\times t}$$

where **Δ*m***_**sample**_ (in kg) is obtained from the mass of the swollen hydrogel before (***m***_**0**_) and after 4 h (***m***_**t**_) of SDD. Note that 2D refers to the top surface area and not to the geometry of the solar evaporator hydrogel, which is tridimensional.

Considering that our system suffers a size change upon heating, the ER values were also calculated considering the top projected area and the lateral wall (**ER**_**3D**_) of the soft material after 4 h of operation time. In this case, eq 2 was applied

2 $${ER}_{3D}=\frac{{\Delta m}_{sample}}{{Area}_{3D}\times t}$$

The evaporation rate (**ER**_**3D**_) is determined by quantifying the mass of water evaporated as explained above, divided by the surface area exposed to direct sunlight (the projected area of the upper surface) and the lateral area (**Area**_**3D**_) of the hydrogel irradiated by reflectance of sunlight in the reflector walls (both in m^2^), per unit of time (***t***, in h). To calculate the area of top and lateral, we considered a conical frustum geometry in eq 3

3 $${Area}_{3D}=\pi r^{2}+\pi \left(R+r\right)l$$

where ***R*** is the bottom radius of the hydrogel; ***r*** is the top radius; and *l* is the slant height (in m^2^). The bottom surface was not considered because it does not receive direct or indirect sunlight and does not affect the interfacial evaporation of water.

Finally, the size change of top exposed surface after SDD is determined by eq 4. The size shrinkage was

4 $$Size\;change\;shrinkage\left(\%\right)=\frac{D_{SDD}-D_{0}}{D_{0}}\times 100$$

where ***D***_**0**_ and ***D***_**SDD**_ are the dimensions of the diameters of the top surface before and after the experiments of SDD (in cm), respectively.

Cyclic SDD with the best composition (highest ER) was carried out by preswelling the hydrogel in seawater for 2 h and executing the solar irradiation over 4 h more. A total of 8 cycles were performed, and the results are discussed in Section 3.4.

### 2.5 Solar-Driven Evaporation Study in a Closed Condensation System (Model IV)

This experiment was conducted with the hydrogels arranged in a “reflector-assisted” configuration (Model III) and with a cover of glass (concave glass lid) to favor condensation of the steam generated. The seawater swelling procedure and SDD timings were also preserved. Thus, the efficiency of the SVG and the material itself was evaluated by the volume of freshwater collected in 4 h. The mass loss and ER calculations were also determined by following the equations described in the previous section. The water quality of the collected water was assessed by using inductively coupled plasma mass spectrometry (ICP–MS).

## 3 Results and Discussion

### 3.1 Preparation and Characterization of Superabsorbent Hydrogels

Targeting to potentiate the use of fully organic, thermosensitive, conducting polymer-based hydrogels for solar-driven interfacial evaporation technology, here we report the ease and affordable synthesis of three PNIPAAm-PAAm/PEDOT/PSS superabsorbent hydrogels, following our previous procedures reported elsewhere.^25,38^ Different molar ratios between NIPAAm and AAm monomers (PNIPAAm-PAAm 1:2, 2:1, and 4:1) were chosen as a proof of concept to demonstrate the impact of hydrogel thermosensitivity in promoting steam production under sunlight. Although the presence of solar absorber components, such as PEDOT/PSS, is a key factor to consider in solar UV absorption technology, we found very good results with a low amount of CP (1 wt %), which will be discussed further. The chemical structures of the different compounds used to prepare the gels are illustrated in Scheme 1a. Figure S1 represents the chemical route followed for the preparation of the PNIPAAm-co-PAAm hydrogels, cross-linked with MBA and converted into photothermal materials after the incorporation of the conducting polymer. Therefore, the cylindrical samples corresponding to control samples (Scheme 1b) were colorless and practically transparent in their aspects, whereas the samples with CP are dark blue due to the presence of the photothermal absorber. In Scheme 2, an illustration of the main SVGs developed until now and the four models used in the present work can be seen to validate the hypothesis of heating losses minimization with a “reflector-assisted” tool (3rd generation). The experimental section includes a detailed architecture description for the different models, as shown in Scheme 2b.

The chemical structure of the TSH hydrogels was evaluated by Raman (Figure 1a,b) and FTIR (Figure S2) spectroscopies since the former technique evidences the less polar groups with stronger intensities than polar groups, whereas the latter is ideal to observe the more intense and sharp absorption bands from polar groups. In the Raman spectra, the presence of C–H stretching peaks was localized in the range 2880–2980 cm^–1^ and confirmed with the CH_2_ and CH_3_ bending (1459 and 1395 cm^–1^) and C–H wagging (845 cm^–1^) bands. Regarding the polar groups in Raman spectra, the most important highlighted was Amide I band (C=O, 1650 cm^–1^) coming from both PNIPAAm and PAAm (Figure 1b) and represented by a broad peak.^38^ The different molar ratios between NIPAAm and AAm units in the feed reactor were confirmed by deconvolution of the Amide I absorption band (inset of Figure 1b). Those bands were also featured in FTIR spectra (Figure S1), with more evidence of amide groups (–CO–NH): N–H stretching with hydrogen bonds interactions at ∼3200–3300 cm^–1^ (Amide A); Amide I at 1635 cm^–1^; Amide II at 1540 cm^–1^; and Amide III at 1170 cm^–1^. In fact, the higher amount of NIPAAm units in PNIPAAm-PAAm (4:1) hydrogel is evidenced by the doublet peak well visualized at 1367 cm^–1^, corresponding to the gem-dimethyl group (–CH­(CH_3_)_2_).^39^ The CP (1 wt %) could not be identified by FTIR spectroscopy; therefore, Raman spectroscopy (Figure 1c) was selected to identify its main functional groups. The peak intensity corresponding to the aromatic thiophene ring (C=C) at 1434 cm^–1^ was practically invariable with respect to the CH_3_ group at 2921 cm^–1^ for the different hydrogel compositions (Figure 1c, inset table). Another absorption band detected was that related to the methylenedioxy units at 991 cm^–1^, which is attributed to the presence of PEDOT.

**Figure 1:**
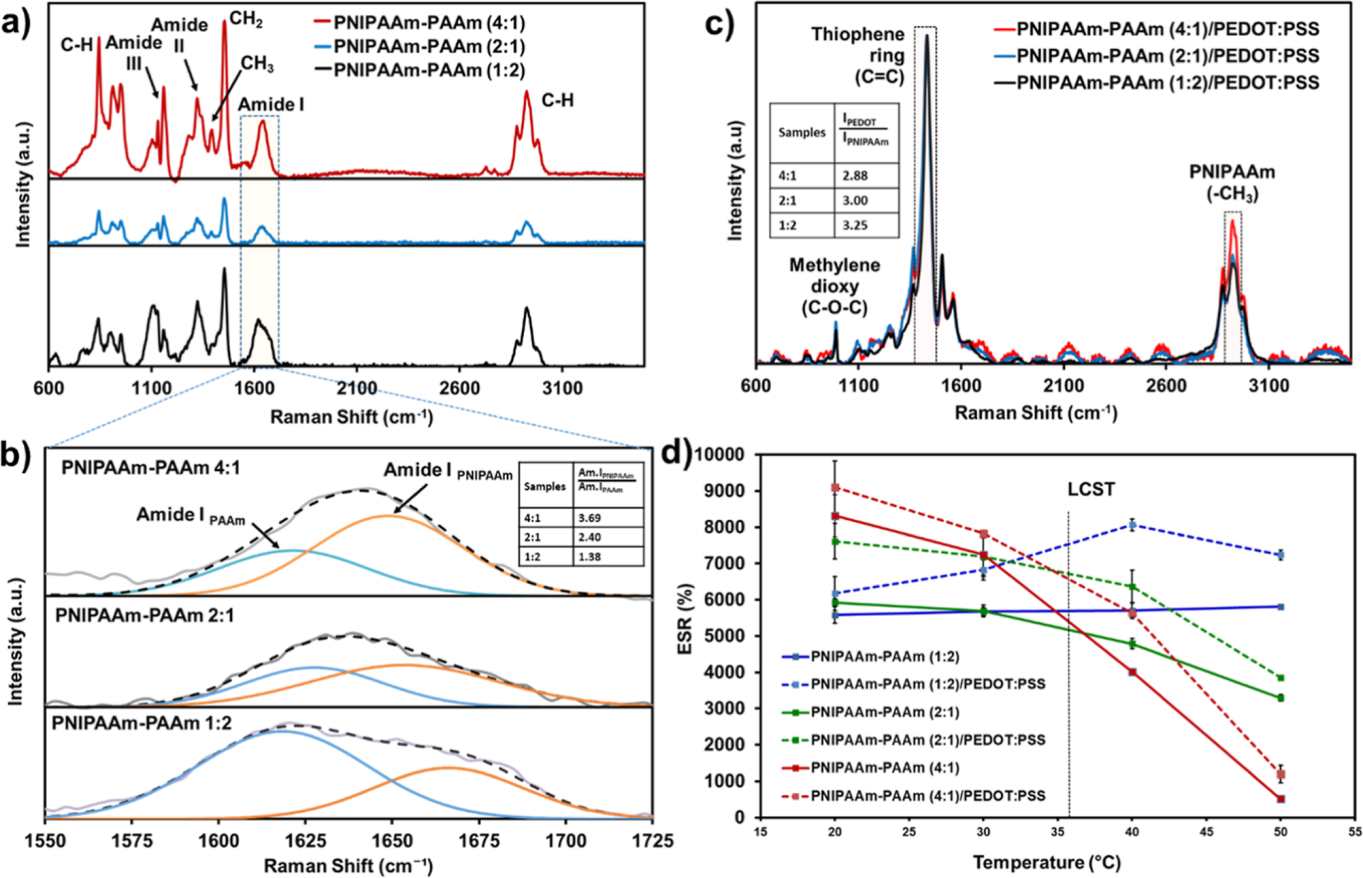
(a) Raman spectra for PNIPAAm-PAAm samples with different NIPAAm/AAm molar ratios and without conducting polymer. (b) Enlarged Raman shift from 1550 cm^–1^ to 1715 cm^–1^ highlighting the Amide I deconvolution of absorption bands ascribed to NIPAAm and AAm segments in the copolymer composition. The table inset shows the molar ratio obtained from the intensity of Amide I absorption band, for each component. (c) Raman spectra of TSHs with conducting polymer, highlighting the presence of both components. (d) Variation of ESR with increasing temperature. LCST refers to the lower critical solution temperature of PNIPAAm-PAAm copolymers.

The thermoresponsiveness of the copolymers was evaluated by monitoring the ESR with temperature in a range close to the lower critical solution temperature of PNIPAAm (LCST, 32–33 °C). It is widely known that the presence of AAm units causes an increase in the LCST.^40^ Indeed, low NIPAAm content with respect to AAm (1:2 molar ratio) had almost constant swelling properties under heating, whereas the molar ratio of 2:1 presented a subtle decrease in water absorbed, either without or with CP (Figure 1d). In contrast, for PNIPAAm-PAAm (4:1) and PNIPAAm-PAAm (4:1)/PEDOT/PSS hydrogels, the raising temperature drastically reduced the water content by 50% from 20 to 40 °C and almost 94% of reduction if compared to the ESR values at 20 and 50 °C. The fast decay occurred at temperatures close to 36 °C, which is correlated to the LCST of this copolymer. Although all compositions presented a very high swelling of water at ambient temperatures (5500–7500% in weight), calculated by eq S1, PNIPAAm-PAAm (4:1) and PNIPAAm-PAAm (4:1)/PEDOT/PSS) samples displayed the best superabsorbent performance with ESRs close to 9000% at 20 °C and the highest water deswelling when the temperature reached 45–50 °C. As described in the next section, this fact influences the ER under sunlight irradiation. In all cases, the presence of only 1 wt % of CP slightly increased the swelling ratio, which we attributed to the presence of highly polar counterions from PSS molecules present in the CP molecules, thus establishing more hydrogen bonds with water. The hydrogen bond interactions of the polymer matrix with water (bound water, BW; intermediate water, IW; and free water, FW) will be discussed later. Finally, the water-retention properties are further confirmed by hydrogel images showing diameter expansion after 24 h of immersion in the seawater solution (Figure S3). The flattened effect of the base seen in the sample does not refer to the “pudding effect” described under sunlight radiation but to the large amount of water trapped inside the gel and the gravity effect always observed for the soft substance.

### 3.2 Hydrogel Morphology, Porosity, and Water Bond Interactions

Porous hydrogels guarantee that the material will have good evaporation performance under solar heating. SEM morphology explorations help on this characterization step (Figure 2). As can be appreciated in Figure 2a–d, the pore diameters decreased when very few amount of PEDOT/PSS was introduced in the polymerization reaction, these results being completely opposite to the trend reported in our previous works with higher proportions of CPs.^25,36^ Based on previous works with CPs, it is known that some interfacial electrostatic interferences with polar groups, like –NH_2_ in AAm monomer, –OH groups in PVA, or –COOH/–COO^–^ in alginate polysaccharides can be responsible for the morphological differences.^41–43^ However, a very high content of NIPAAm monomer, with hydrophobic isopropyl groups (i.e., composition 4:1), led to a decreased and mostly equal pore size distribution with the inclusion of PEDOT/PSS CP (Figure 2e,f). The similar porosity between PNIPAAm-PAAm (4:1) and PNIPAAm-PAAm (4:1)/PEDOT/PSS was confirmed by micro-CT (Figure 2g,h). As a matter of fact, either the hydrogel without or with PEDOT/PSS component showed almost equal porosity (88% and 85%, respectively). Nevertheless, the porous inner structure is characterized by an irregular pore size distribution, and the highest number of pores have pore sizes of 42 μm for both samples, which are values much larger than those obtained by SEM topography. The presence of AAm units caused a “stick” effect on soft gels, and after the lyophilization process, some pores decreased in size or flattened.^38^ This effect was more pronounced in the outer layers, which were the ones observed by SEM. Therefore, micro-CT revealed more reliable values about the bulk porosity of the samples in the solid state. The limitations of pore size measurements using samples previously freeze-dried have been elsewhere reported.^44^ Even if this is strongly affected by the strength of the cross-linking network, the observed pores in SEM images could be not intrinsic to the native gel matrix because they are generated during the freeze-drying process. Herein, the same standard protocol has been applied to all of the samples investigated in order to minimize the deviations and ensure a correct qualitative comparison of the internal structure of hydrogels.

**Figure 2:**
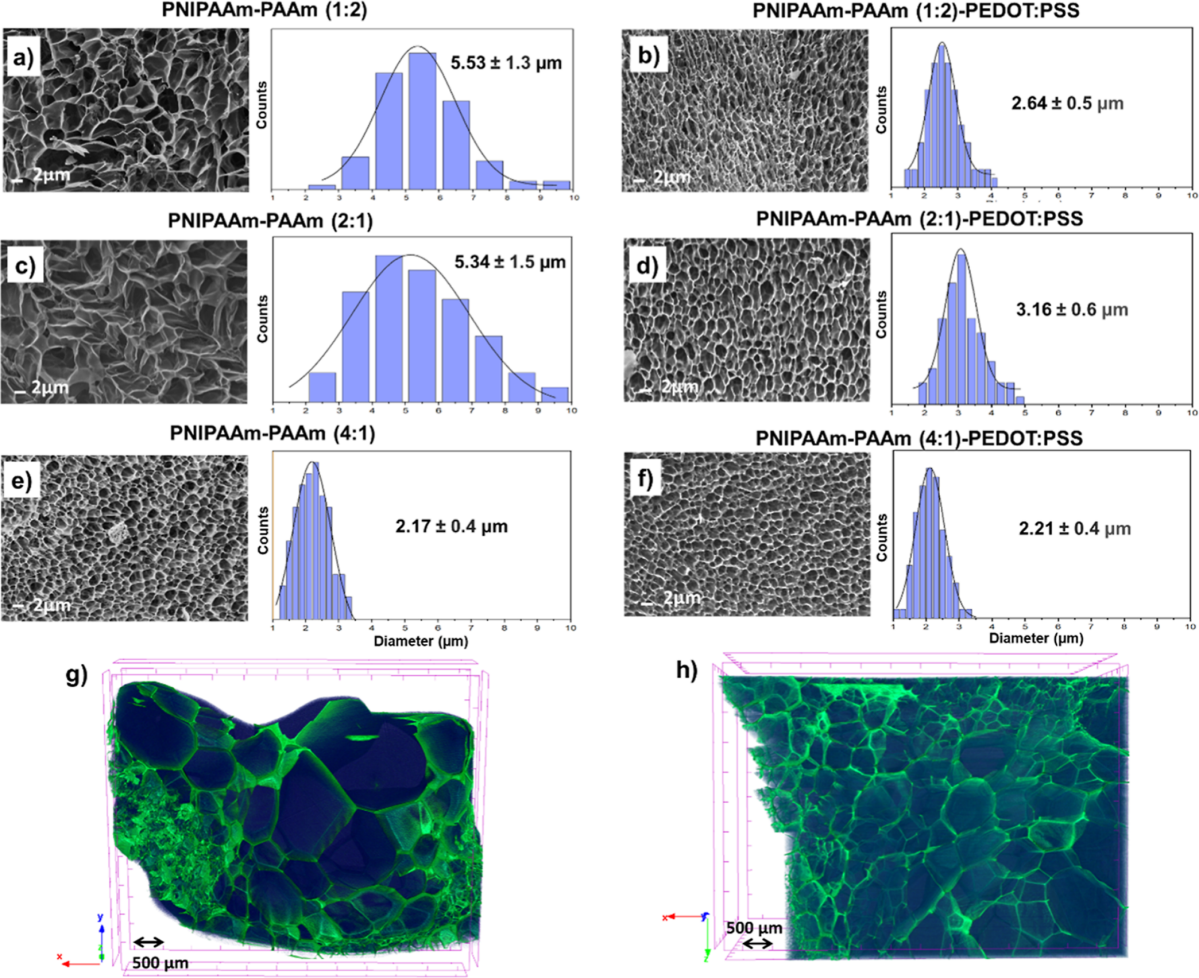
SEM topography and pore size distribution of: (a) PNIPAAm-PAAm (1:2); (b) PNIPAAm-PAAm (1:2)/PEDOT/PSS; (c) PNIPAAm-PAAm (2:1); (d) PNIPAAm-PAAm (2:1)/PEDOT/PSS; (e) PNIPAAm-PAAm (4:1); and (f) PNIPAAm-PAAm (4:1)/PEDOT/PSS. Scale bars represent 2000× of magnification for all samples. Pore sizes and standard deviations were obtained from the maximum peak of Gaussian curves. Micro-CT images show the inner porosity of TSH hydrogels in solid foam states (lyophilized): (g) PNIPAAm-PAAm (4:1) and (h) PNIPAAm-PAAm (4:1)/PEDOT/PSS samples.

Although porosity is mandatory for efficient solar-driven water evaporation, pore sizes and swelling capacity are not interdependent properties; i.e., bigger pores do not imply higher swelling ratios. According to previous literature, hydrogen bond interactions play a more crucial role on the ESR and, in turn, in the interfacial water steam generation. Conversely, some authors stated that hydrophilicity–hydrophobicity properties affect substantially the performance of water vapor steam production.^7,45^

In our model, the superabsorbent and thermosensitive properties toward thermal heating promoted by the sun are necessary to reach higher ERs. PNIPAAm consists of two parts: hydrophilic (–NHCO–) and hydrophobic (–CH­(CH_3_)_2_) segments, whereas PAAm remains mostly hydrophilic thanks to the primary amide pendant group.^46^ Such polar groups establish numerous hydrogen bonds with water. To boost the evaporation process, the enthalpy associated with this phase transition has to be minimized and, in this direction, a good design of the chemical structure of the material plays a crucial role.^45^ The last statement comes from the theory supported by several studies, which show that the vaporization enthalpy is strongly connected to the interaction of water molecules with the surface of the material, being meaningful to the kind of linkages they establish.^2,47^ Hence, depending on the nature of bonding, it is possible to distinguish three types of water molecules: (i) free (FW), similar to pure water and totally free from interactions with polymer chains, (ii) bound (BW), able to form hydrogen bonds with hydrophilic groups in polymers, and (iii) intermediate (IW), which interacts weakly with the polymer and has an intermediate structure between bound and free water.^25,48^ Among these different states of water molecules, several authors already demonstrated that IW present the lowest vaporization enthalpy because of the kind of bonds established [5]. Prompted by this fact, a good strategy to improve the evaporation performance corresponds to the insertion of functional groups that can create IW in the hydrogel backbone.

To better understand the kind of water present in the PNIPAAm-PAAm copolymers, the relative amount of IW and FW was calculated using the deconvolution of –OH stretching region, related to hydrogen bonds, with Raman spectroscopy (Figure 3). This technique allowed us to clearly distinguish between FW and IW, while the strong interactions of BW are hindered due to the signal obtained from IW and FW. The broad band located in the range 2800–3800 cm^–1^ attributed to the –OH stretching region of water molecules has been deconvoluted into four main peaks related to different hydrogen bonding patterns of water molecules with the polymer backbone. The peaks located at 3197 cm^–1^ and 3317 cm^–1^ correspond to FW with four hydrogen bonds, while the peaks located at 3457 cm^–1^ and 3624 cm^–1^ are associated with weakly hydrogen-bonded IW.^25^ Band deconvolution revealed that an almost equal proportion of IW/FW was present in all samples without PEDOT/PSS (Figure 3a and inset). The values obtained varied from 0.87 to 0.89 and 1.05 for PNIPAAm-PAAm (1:2), PNIPAAm-PAAm (2:1), and PNIPAAm-PAAm (4:1), respectively. With the incorporation of the CP (1 wt %) such proportions increased. As shown in Figure 3b, the Raman shift of the four peaks was constant, with some small shift toward higher values only for FW (from 3197 to 3209 cm^–1^ for FW_1_, and from 3317 to 3319 cm^–1^ for FW_*2*_). More marked differences were observed in the variations of the IW/FW ratio that in all cases investigated is higher than 1, which indicated a bigger proportion of IW with respect to FW in all samples. Indeed, the electrostatic interactions offered by ionic polymers as PSS favored the formation of hydrogen bonds that are responsible for the increased amount of IW detected by Raman. As reported in the inset of Figure 3b, the IW/FW ratio increased from 1.09 to 1.40 and 1.62 for PNIPAAm-PAAm(1:2)/PEDOT/PSS, PNIPAAm-PAAm(2:1)/PEDOT/PSS, and PNIPAAm-PAAm(4:1)/PEDOT/PSS, respectively. Through these analyses, we identified that a greater quantity of acrylamide does not correspond to an increase in IW but rather translates into a decrease in FW present in the system. This effect is attributed to the formation of more BW which, as previously demonstrated, could also act as a cross-linker between the polymer chains, thus affecting the diffusion of water molecules inside the system and, therefore, the evaporation process.^49^ In conclusion, the CP induced some effect on these properties, which can be further magnified by the amount of NIPAAm units.

**Figure 3:**
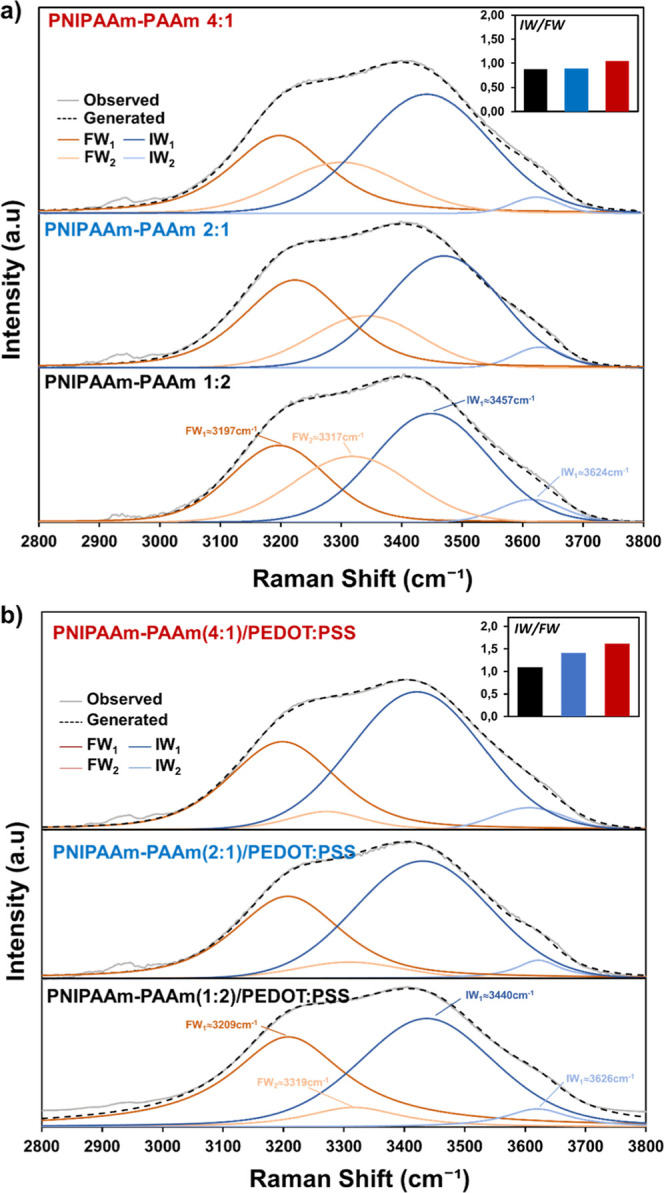
Raman spectra deconvolution curves in the zone related to the hydrogen bonding interactions of water with amide groups of: (a) PNIPAAm-PAAm (1:2), PNIPAAm-PAAm (2:1), and PNIPAAm-PAAm (4:1); and (b) PNIPAAm-PAAm (1:2)/PEDOT/PSS, PNIPAAm-PAAm(2:1)/PEDOT/PSS, and PNIPAAm-PAAm (4:1)/PEDOT/PSS samples. Intermediate-to-free water ratios (IW/FW) are shown as inset bar plots.

### 3.3 UV Absorption and the Mechanical Deformation of Superabsorbent, Thermoresponsive, and Solar Absorber PNIPAAm-PAAm/PEDOT/PSS Hydrogels

In addition to an internal structure interconnected by pores, the hydrogels must display mechanical stability for cyclic tests and upcycling during solar evaporation, and the material itself should have high absorptivity in the UV–vis spectrum, so the copolymers should be able to absorb the maximum solar power. In this sense, PEDOT/PSS has been uniformly integrated as a photothermal promoter into PNIPAAm hydrogels.^25,36^ As observed in Figure S4, the dark material (with CP) absorbed almost 90% of light in the visible region, whereas white gels let the radiation pass across them (10–20%).

In SDD, the hydrogels could be used several times in batch or continuous mode. Hence, its mechanical performance needs to be carefully evaluated. First, uniaxial compression testing was conducted on cylindrical hydrogels at a compression rate of 1 mm min^–1^ until a strain of 70% was reached, and all samples were recovered without breaking. The elastic moduli and compressive strength values, which were determined from single stress–strain curves (not shown), are included in Table S1. From sample manipulation, hydrogels with a higher NIPAAm component were stiffer than the other two formulations since AAm units are much more flexible and malleable.^38,50^ This observation was verified with quantitative data, as PNIPAAm-PAAm (4:1) and PNIPAAm-PAAm (4:1)/PEDOT/PSS hydrogels displayed the highest Young’s moduli (2.34 ± 0.24 and 2.48 ± 0.38 kPa, respectively; Figure 4b). Besides, the addition of the CP did not affect the compressive strength of the 4:1 formulation, which remained high at ca. 11 kPa. Later, cyclic compression testing (5 cycles) was carried out to evaluate the mechanical integrity of the samples under deformation (Figure 4a). In Figure 4c,d, the images of PNIPAAm-PAAm (4:1)/PEDOT/PSS and PNIPAAm-PAAm (4:1)/PEDOT/PSS samples before and after the first cyclic test can be visualized. As can be seen, both samples hold their cylindrical form after release. In Figure S5, the first and fifth cycles of compression tests are represented, and in Figure S6, photographs similar to those of Figure 4c,d, is also shown for the other compositions for comparison. Table S1 summarizes the values extracted from the cyclic testing. The highest hysteresis was shown by the sample with four times more molar ratio of NIPAAm than AAm (Figure 4a).

**Figure 4:**
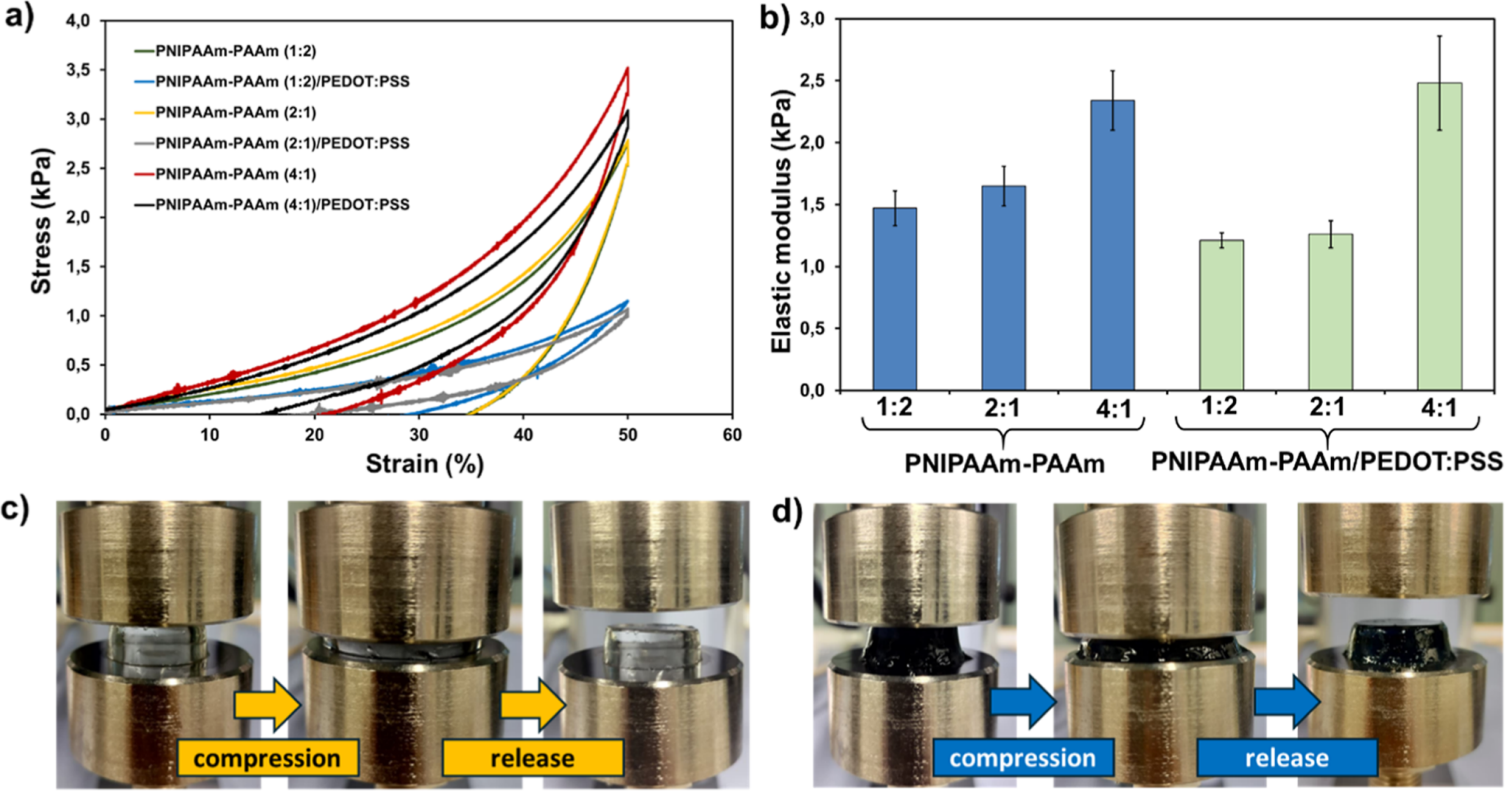
(a) Cyclic compression tests representing the first cycle of all samples analyzed; (b) comparison of elastic modulus obtained from the first compression cycle; (c,d) photographs from PNIPAAm-PAAm (4:1) (c) and PNIPAAm-PAAm (4:1)/PEDOT/PSS and (d) hydrogels showing the compression and release of samples in cyclic tests.

The incorporation of the CP resulted in a consistent decrease in hysteresis loss (HL) across all tested hydrogels, which will be beneficial for their necessary reutilization (cyclic experiments or continuous use) in SDD tasks. Indeed, in the first compression cycle, all PEDOT-containing hydrogels exhibited lower energy dissipation (H, kJ/m^2^), as well as HL (%) compared to the hydrogels without CP. We attribute this reduction to the reinforcing effect of PEDOT/PSS, which introduces additional physical cross-links, such as hydrogen bonding and electrostatic forces, thus facilitating stronger intermolecular interactions between the CP and the PNIPAAm-PAAm hydrogel matrix.^51^

Among all synthesized formulations, the PNIPAAm-PAAm (4:1)/PEDOT/PSS hydrogel demonstrated superior fatigue resistance, with the lowest HL in the first cycle (42.0%) and the smallest reduction in hysteresis over five cycles (Δ*H* = – 35.15%). In contrast, the 1:2 formulation exhibited the highest hysteresis drop (Δ*H* = – 86.38%), which reflects extensive internal rearrangement and mechanical degradation. The higher PNIPAAm content in the 4:1 hydrogel contributes to a stiffer, more elastic network capable of dissipating less energy per cycle and recovering its structure under repeated stress.^52^

### 3.4 Solar-Driven Evaporation in Open Air Comparing Three Models of SVG Arrangements

Considering the superior swelling properties and mechanical features of the PNIPAAm-PAAm (4:1)/PEDOT/PSS hydrogels, this composition was chosen for the SDD experiments with the three SVG models designed for the present study (Figure 5a–c). In the case of the hydrogel enclosed in an insulating foam and in contact with cold water (Model I, Figure 5a), the system was able to reach a steady-state temperature of 34 °C (*T*_<LCST_) in approximately 90 min (Figure 5d, *t*_s_). Nevertheless, it was not enough to overpass the copolymer LCST temperature (∼36 °C). Therefore, the 3D structure was not able to concentrate all the energy input of 1 sun. The energy losses by dissipation to the cold-water underneath were the main reason for the lower mass loss (7.17 kg·m^–2^, Figure 6a) observed with the Model I device (Figure 5a), as also highlighted by other researchers.^3,53^ Thus, in this model of evaporator assembly, the TSH was not able to shrink enough to offer the “pudding effect” we unveiled in our previous studies with PNIPAAm/PEDOT and ALG-PNIPAAm/PEDOT.^25^ In contrast, the reflector-contained model (Model II, Figure 5b, recently reported by Liu and co-workers^35^), showed much higher efficiency in terms of water mass loss over time. In only 30 min, the superabsorbent gel was able to hold out a *T*_>LCST_ of 40 °C (Figure 5e), thanks to the positive environmental energy input received from the metallic container (reflectance effect). In 4 h, the hydrogel has been contracted and expulsed enough water to offer a mass loss of 18.91 kg·m^–2^ (Figure 6a) and an ER_2D_ of 4.73 KMH (Figure 6b), almost three times superior to the Model I (1.79 KMH).

**Figure 5:**
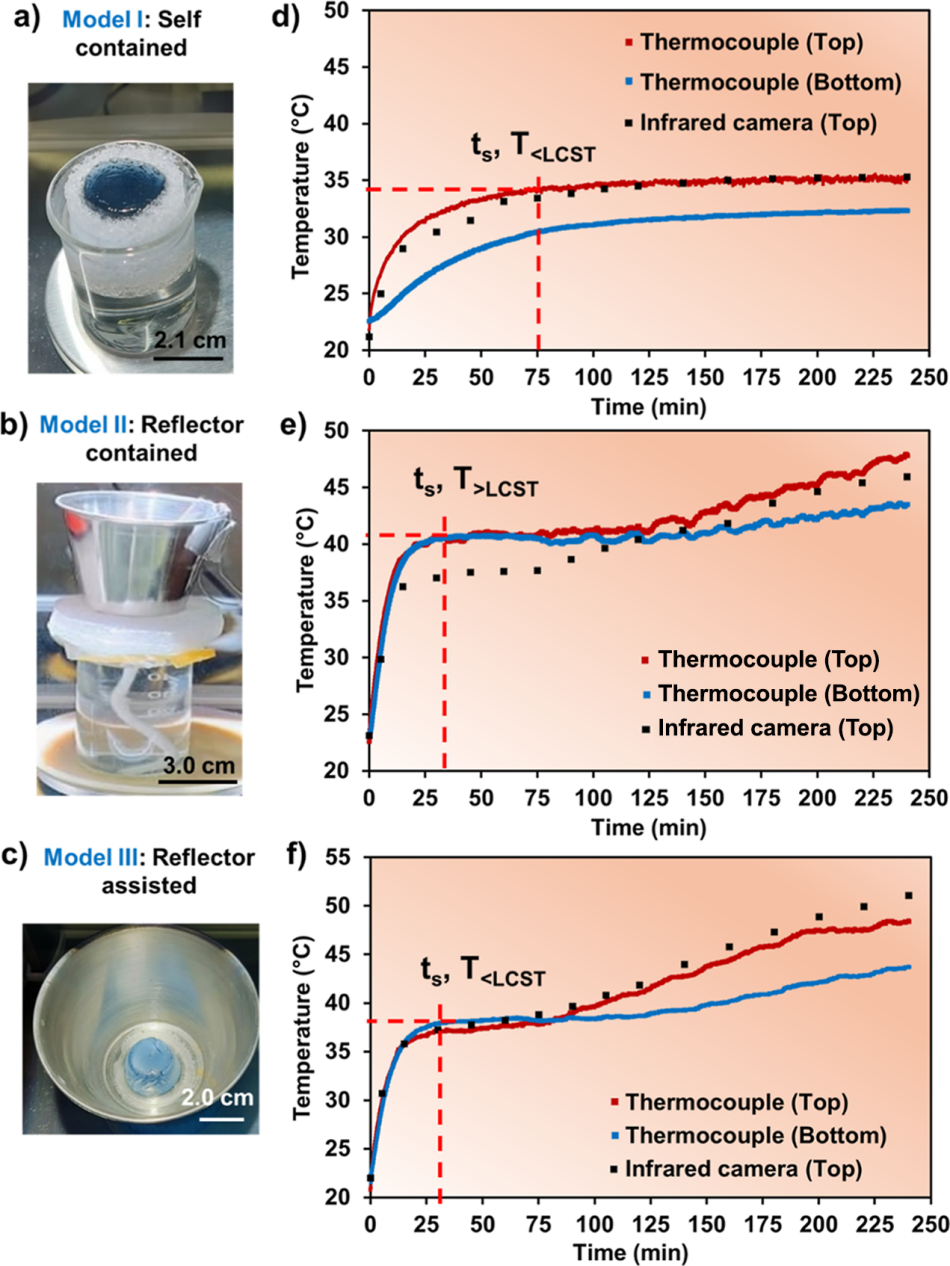
(a–c) Photographs of the three evaporator models with PNIPAAm-PAAm (4:1)/PEDOT/PSS samples before SDD assays. (d–f) Temperature profiles with time by using two thermocouples (placed in the bottom and in the top of hydrogel surfaces) and an IR camera (top surface calibrated), where *t*_s_ refers to the time of reaching a steady state (or plateau) and *T* refers to the temperature reached at that *t*_s_, which can be below or higher than the hydrogel copolymer LCST (∼36 °C).

**Figure 6:**
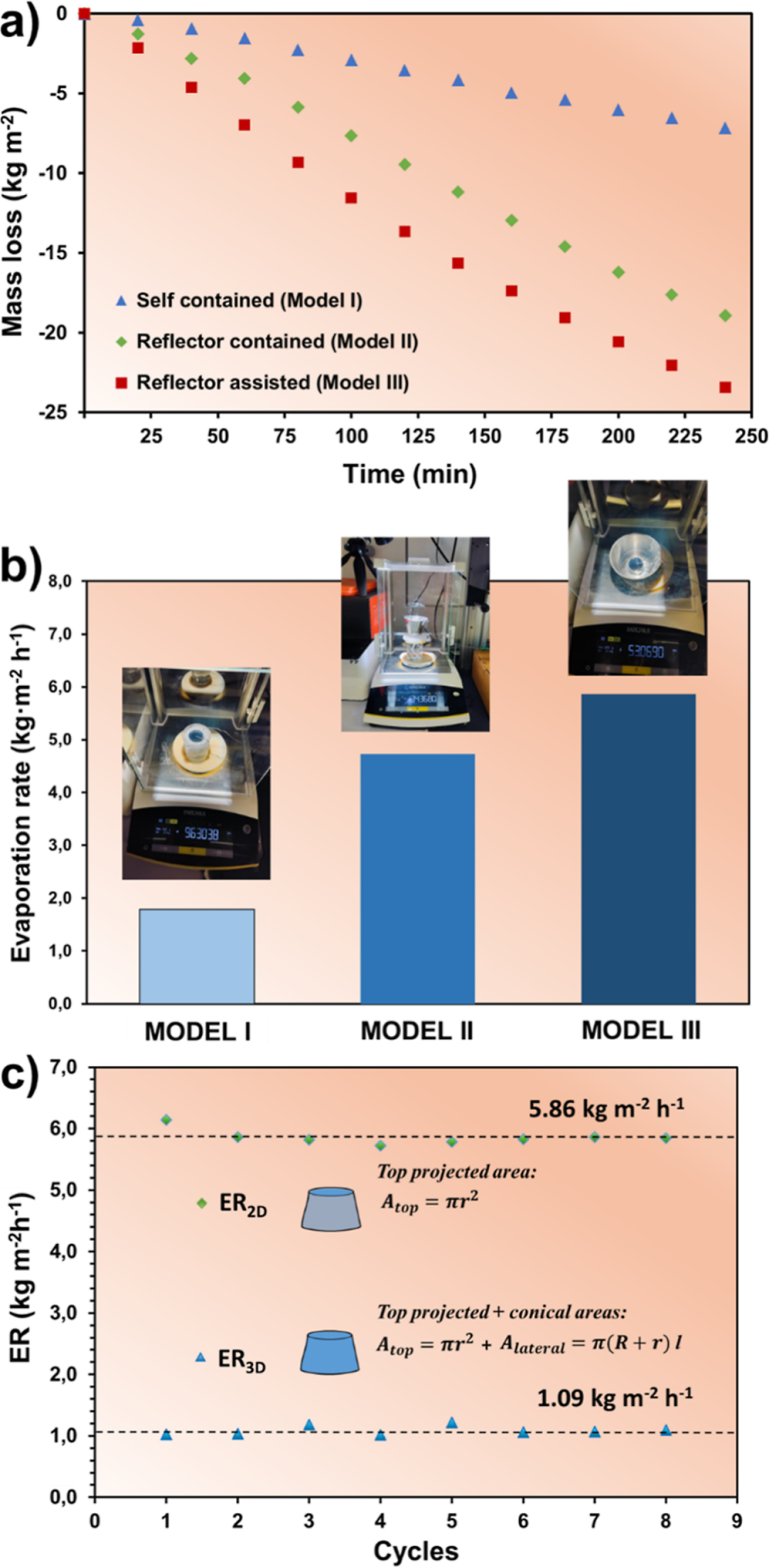
(a) Mass loss of swollen hydrogels with increasing sunlight irradiation comparing the 3 evaporator models. (b) Evaporation rate (ER_2D_ calculated from eq 1), for PNIPAAm-PAAm (4:1)/PEDOT/PSS hydrogel in SDD experiments with the 3 evaporator models showed in the inserted photographs. Although some replicates were performed, only the best values from one sample are expressed in plots a,b. (c) ER_2D_ and ER_3D_ (calculated from eqs 1 and 2) of PNIPAAm-PAAm (4:1)/PEDOT/PSS hydrogel after 8 consecutive cycles of water evaporation under 1 sun. Each cycle corresponds to the ER of the same sample after 4 h and hydrogel reswelling in seawater, demonstrating the potential of this hydrogel for reusability.

In the third example, reflector-assisted (Model III), the design represents a system without continuous input of salt water, contrary to the previous cases, which means that energy losses by thermal conductive transfer from our material to the water (across the foam or the wick, Scheme 2b) were not expected. Following the thermal profile presented in Figure 5f, we observed that high temperatures (*T*_>LCST_ = 37.9 °C) were also achieved in a faster way (∼30 min) than in Model I. The concentration of 1 sun power projected to the top surface and to the lateral walls (by reflectance irradiation assisted by the metallic accessory) pushed the steam production until a mass loss of 23.4 kg·m^–2^ (Figure 6a). The shrinkage of the top surface, calculated from eq 4 was at about 30–33%, which led to an ER_2D_ of 6.05 KMH (Figure 6b). Considering that the lateral surface is also receiving part of the solar light, this area has been also introduced in the calculation of the ER, now defined as ER_3D_, as reported in Table 1. The ER_3D_ drastically decreases when compared to ER_2D_, and it is not representative of the large amount of water recovered herein. The rationale for preferring ER_2D_, instead of ER_3D_, is based on both experimental observations and several studies in the literature about SDD from hydrogels. Actually, there is a consensus among the scientific community to consider only the top projected area as the unique zone with a fixed 1 sun power impact. It is correct to consider that, especially when the solar evaporator is coupled with a reflector, the lateral surface could function as an evaporation surface, being the light scattered from the inner wall of the reflector toward the lateral surface of the 3D hydrogel. However, in this specific case, the reflection of the light mainly benefits the achievement of temperatures difficult to obtain in the absence of it, but the angle employed (70°) does not favor the evaporation from the lateral surface, as previously reported by Liu and co-workers.^35^ In addition, the conic geometry achieved because of the thermoresponsiveness of the materials contributes to increasing the angle between the reflector and the lateral surface of the hydrogel, further decreasing the contribution of the lateral surface to the evaporation process. As proof, infrared thermal images for Model II are inserted in Figure S7 for comparison. It shows that the lateral surface temperature is lower than the ambient temperature, allowing heating interchange with the environment. As a conclusion, the environmental energy input is added to the solar energy received directly on the top surface, improving the energy content of the system, without increasing the real evaporation surface. In this regard, the top surface is considered the unique part of the system where the water evaporation takes place, and as a consequence, ER_2D_ fits better the real performance, if compared to ER_3D_.

**Table 1:** Calculations of ER Based on the Hydrogel Projected Top Surface Area (ER_2D_) and from the Effect of Reflector Wall Irradiation on the Lateral Structure, Considering That Similar Sunlight Energy Reached Both the Top and the Lateral Wall (ER_3D_) for PNIPAAm-PAAm (4:1)/PEDOT/PSS Hydrogel, Employing Model III Cell Construction

	model III: open-air system						external inputs
sample n°	mass loss (×10^–3^ kg)	area of top (×10^–4^ m^2^)	total area (top + lateral) (×10^–4^ m^2^)	size change shrinkage (%)	ER_2D_ (kg·m^–2^ h ^–1^)	ER_3D_ (kg·m^–2^ h ^–1^)	RH^a^(%) RT^b^(°C)
1	4.7112	2.00	10.56	30.43	5.85	1.11	57.80%
2	5.0183	2.00	10.56	33.33	6.23	1.18	26.4 °C

a Notes: RH: Relative humidity.

b RT: Relative temperature.

Although the material cannot reach a thermodynamic equilibrium, it is quite interesting that the TSH achieved stable dimensions after solar evaporation. The top surface contracted to a limit of 1.6 cm (*D*_SDD_, in eq 4), independent of the variable diameter obtained after the seawater swelling for 2 h (*D*_0_, 2.3–2.8 cm), and the height reduced to 1.5 cm (from an initial value of 2.0 cm). Thus, as can be seen in Table 1, the mass loss (Figure 6a) was the predominant register that influenced the solar evaporator performance. Moreover, in Model III, the mass loss was related uniquely to the quantity of water trapped in 2 h inside the gel. Accordingly, fluctuations of mass weight due to the continuous water flow from an external source were not possible.

In summary, Model III yielded the highest ERs values, thus confirming that the metallic tool is a powerful alternative to the classical “self-contained foam” devices, even if thermodynamic equilibrium cannot be achieved. Moreover, PNIPAAm-based TSH expelled water in a faster way when the thermal temperature was higher than its LCST (∼36 °C, for this copolymer), also proving that the change of the cylindrical form to the conical frustum format, after solar irradiation, is beneficial. Additionally, multiple evaporation-swelling cycles were carried out to test the mechanical stability of PNIPAAm-co-PAAm (4:1)/PEDOT/PSS in seawater. Figure 6c compares the ER considering the top surface (as the unique projected area of sunlight irradiation), and that of the three-dimensional configuration, considering the conical lateral surface and the top. The hydrogel maintained an almost constant ER throughout all eight cycles, exhibiting a strong shape-recovery capability and high elasticity. These properties allow it to retain the same performance across all cycles, demonstrating a stable operation and confirming its reusability for repeated SDD processes.

As very recently reported,^54^ the achievement of high-performance in water evaporation is due to a combination of interfacial interactions, as the hydrophilic matrix is able to increase the IW molecule content, the surface contraction, and good water transport capacities (porous structure and water flow capillarity), which ensure continuous and appropriate amount of water delivered to the evaporation interface. The exclusive characteristic of PNIPAAm as a thermosensitive material allows a pore reduction above the LCST, which enhances the water diffusion by promoting a faster capillary flow into the three-dimensional pores, if compared to the slower molecular diffusion mechanism. Therefore, by combining thermosensitive hydrogels with a powerful solar evaporator architecture, we have been able to maximize water steam production.

Having established Model III as the most efficient solar evaporator assembly, some considerations are necessary. We should be aware that the ERs (and consequently η) calculated from the top projected area cannot be compared with other references, as claimed in numerous publications because these systems were evaluated with neither the same SVG nor equal environmental conditions (RH, material dimensions, and time of seawater swelling) as ours. This is particularly critical for thermosensitive materials because the system is not able to reach a thermodynamic equilibrium, as evidenced by the constant temperature increasing with the reflector tool (Figure 5e,f). As a consequence, the evaluation of the η in unsteady state thermodynamic systems, as those herein reported, is quite complex and susceptible to errors. Nevertheless, for the sake of comparison within the SDD field, Figure S8 reports the dynamic η and ER (both ER_2D_ and ER_3D_) calculated for the PNIPAAm-PAAm (4:1)/PEDOT/PSS sample, where dynamic refers to progressive observations over time. Three main intervals have been individuated, before and after the thermoassisted volumetric shrinkage of the hydrogel (Figure S8a). Partial (variation of the area, ΔArea = 30.4%) and full contraction (ΔArea = 55.6%) of the hydrogel surface has been observed after 1 and 3 h from the beginning of the experiment, respectively. The η has been calculated by using ER_2D_ values related to each time interval and a proper value of enthalpy, obtained by dark room experiments explained in the Supporting Information (section 2.3). As expected, taking into account the nonequilibrium state and the impressive performance of the materials investigated, the efficiencies observed overpass 100%, especially after the full contraction of the surface area. Figure S8b shows the divergence between ER_2D_ and ER_3D_ over time. Quite interestingly, these values are pretty similar at the shortest times, confirming that the contribution of the lateral surface is negligible and that the higher values of ER_2D_, if compared to ER_3D_, are ascribed to the thermoresponsiveness of the material.

### 3.5 Solar-Driven Evaporation and Water Quality Assessment for Model IV (Closed System with “Reflector-Assisted” Configuration)

Taking into account the positive results from the reflector-assisted evaporator design to promote water mass loss (i.e., water vapor production), we decided to test a fourth model where the PNIPAAm-PAAm (4:1)/PEDOT/PSS hydrogel was evaluated with a condenser (a glass dome lid) above the metallic reservoir (Figure 7a). In this new evaporator assembly, the mass of the hydrogel and its dimensions (Figure 7b) were determined before and after 4 h of solar irradiation. The SDD efficiency of this superabsorbent and photothermal material was determined by the condensed water volume collected with the help of the glass dome lid represented in Figure 7c. With this new model (called “Model IV”) and the reflector assisted tool, the mass losses and ERs (Table 2) were very similar to the open-air experiments (Table 1), sustaining that the process is feasible and reproducible in closed systems. Using this system, the volume of freshwater accumulated was around 2.4–2.5 mL·h^–1^, which is adequate for a soft material with such low dimensions. It can be attributed to the very high swollen mass (10–12 g, respect to 1.3–1.4 g in the dried state) and fast ER. The main advantages of this system are the easy material obtaining, its fully organic composition free of CRMs, and their very low amounts of solar absorbers. Such hydrogels can be prepared in only two steps of synthesis, with very mild conditions, and can be reused several times, by reswelling the pieces in seawater, with careful to not break the gelatin-like format they have. Deng and co-workers^55^ developed an integrated system for seawater desalination and electricity generation with a modular device, having demonstrated it is possible to maximize the potable water production by using 3D hydrogels in serial, similar to solar cell technology. However, the main disadvantage was related to the loss of thermal efficiency in open-air devices and the risk of hydrogel complete drying, if compared to Models I and II, with continuous seawater input.

**Figure 7:**
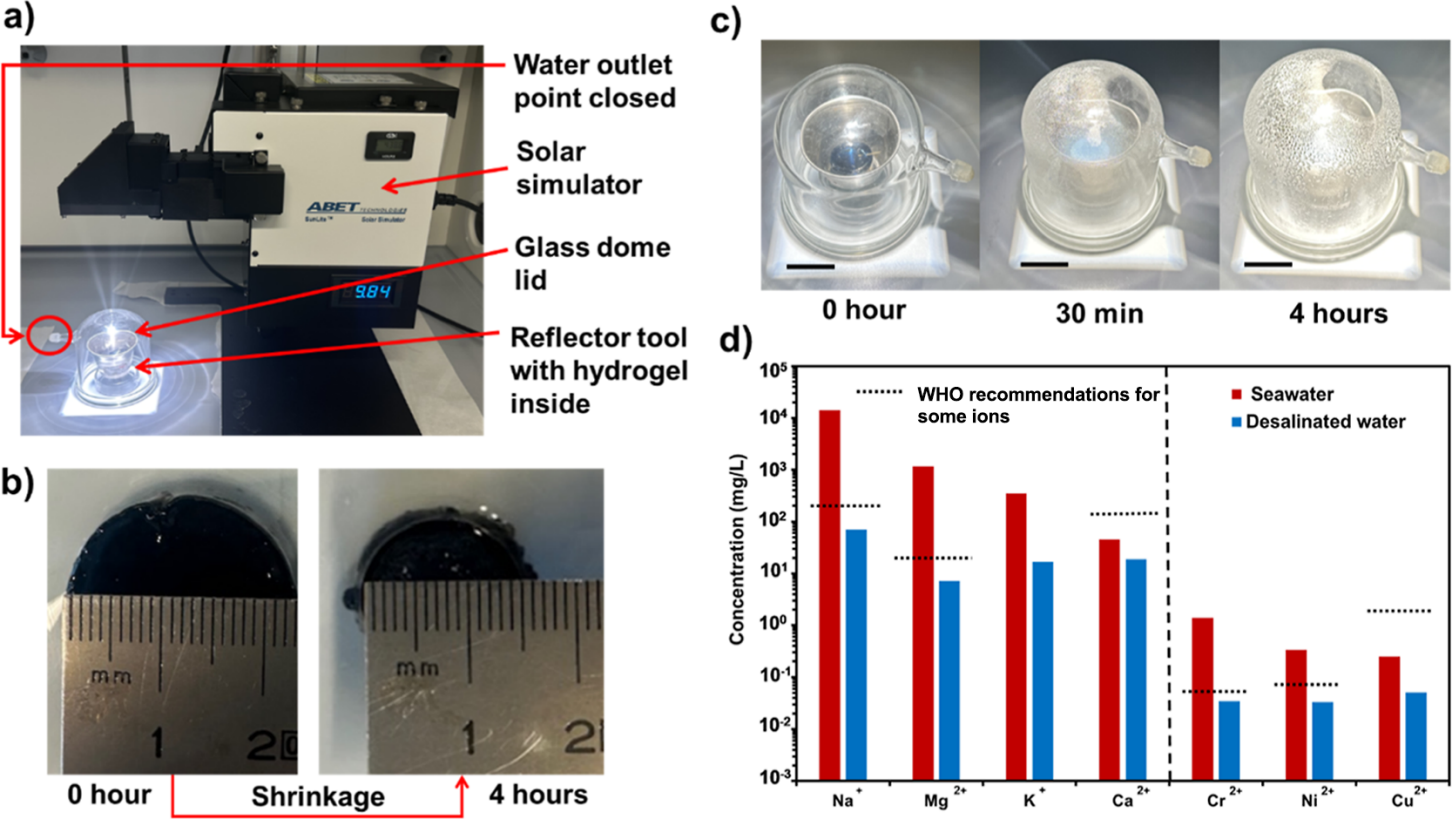
(a) Scheme of SVG assembly (called Model IV) with the hydrogel positioned inside the metallic reflector tool and both covered with a glass dome lid for the collection of condensed water. (b) Photographs of PNIPAAm-PAAm (4:1)/PEDOT/PSS hydrogel before and after solar irradiation for 4 h, showing the “pudding effect” of gel contraction. (c) Evolution of water droplets formation on the top of the glass dome lid with increasing time. Scale bar: 3 cm. (d) Chemical composition of monovalent and divalent cations in seawater (mother solution) and that of the water collected after the SDD assays.

**Table 2:** Calculations of ER Based on the Hydrogel Projected Top Surface Area (ER_2D_) and from the Effect of Reflector Wall Irradiation on the Lateral Structure, Considering That Similar Sunlight Energy Reaches Either the Top and the Lateral Wall (ER_3D_), for PNIPAAm-PAAm (4:1)/PEDOT/PSS Hydrogel Under Solar Irradiation for 4 h, Employing Cell Construction of Model IV

model IV: closed system							external inputs
sample n°	mass loss (×10^–3^ kg)	area of top (×10^–4^ m^2^)	total area (top + lateral) (×10^–4^ m^2^)	size change shrinkage (%)	ER_2D_ (kg·m^–2^ h ^–1^)	ER_3D_ (kg·m^–2^ h ^–1^)	RH (%) RT (°C)
1	4.6643	2.00	10.56	36.00	5.79	1.10	58.90%
2	5.5489	2.00	10.56	31.91	6.89	1.31	27.5 °C

After water droplet recovery, the water quality was assessed by ICP–MS to quantify the concentrations of major cations and heavy metals after the SDD in a closed system. As shown in Figure 7d, the hydrogel demonstrated high removal efficiency for both salt ions and heavy metals. Sodium (Na^+^) concentration decreased from 14092.4 mg L^–1^ to 69.9 mg L^–1^, achieving a removal efficiency of 99.5%. Similarly, magnesium (Mg^2+^) was removed with 99.3% efficiency (from 1172.6 to 7.3 mg L^–1^), potassium (K^+^) with 95.2% (from 350 to 16.9 mg L^–1^), and calcium (Ca^2+^) with 58.7% (from 45.3 to 18.7 mg L^–1^). The hydrogel also exhibited a strong removal capacity for divalent heavy metals. Chromium (Cr^2+^) decreased by 97.5%, nickel (Ni^2+^) by 90.2%, and copper (Cu^2+^) by 80.1%. The final concentrations of monovalent, divalent, and heavy metals were well below the World Health Organization guidelines for safe drinking water.^56,57^ There are currently no specific limits for potassium ions (K^+^).

These results confirm that the PNIPAAm-PAAm (4:1)/PEDOT hydrogel can effectively remove a wide range of ions from seawater. Its high desalination efficiency is attributed to the synergistic effect of the dual-network hydrogel structure and the conductive PEDOT component, which enhances ion capture and transport within the polymer matrix.

## 4 Conclusions

Undoubtedly, in terms of design, we can affirm that the “heating architecture” composed of metallic and reflectance containers favors freshwater steam production. The “reflector-assisted” evaporator revealed an evaporation capacity of 6.04 kg m^–2^ h^–1^ and 6.35 kg m^–2^ h^–1^ in open and closed configurations, which are six times superior to classical first and second solar evaporator device generations. The superabsorbent PNIPAAm-PAAm (4:1)/PEDOT/PSS hydrogel has been proven to be an efficient SVG system for the purification of seawater in all of the models tested. The presence of: (i) a mirror reflectance container, composed by stainless steel; (ii) a highly thermal conductor material (metallic-based, proposed by Liu and co-workers, versus glass conventional reservoirs); and (iii) the lack of direct contact of the hydrogels with the cold seawater solution (proposed in this work) avoids convection and conduction thermal losses from the material to the environment while promoting the start of vapor generation by fast temperature increasing. Thus, this batch process configuration for SDD maximizes the water mass loss and solar-driven efficiencies.

Moreover, one important lesson learned is that a solar absorptivity of 99% is not essential to achieve adequate evaporation performances, with the device architecture being the most relevant. Therefore, greater amounts of solar absorbers in the hydrogel formulation (PEDOT/PSS 1 wt % in our case) can be suppressed. It represents an advantage with respect to systems that incorporate carbon-based powders, chromogenic compounds, or metal oxides, which sometimes require high concentrations of solar absorbers, making them incompatible with the PNIPAAm hydrogel. In today’s world, where new and sustainable technologies for desalination are gaining ground, the SVG and the materials employed in the present study are considered safe by design, since they are completely free of CRMs and they can be reused in SDD cyclic experiments due to their great capacity for water uptake and their excellent mechanical and dimensional stabilities. Future work will focus on extrapolating the model’s IV performance in outdoor environments with natural sunlight exposure.

## Supplementary Material



## Notes

Detailed description of the procedure used for the physical-chemistry characterization of the materials (PDF)
