# Supplementary material for: Unveiling the Challenge of Evaporator Design in Clean Water Production Promoted by Superabsorbent Hydrogels and Sunlight
Source: ACS Appl Mater Interfaces. 2026 Jan 5;18(1):3204–18. doi: 10.1021/acsami.5c20819 (PMC12781103; doi:10.1021/acsami.5c20819)
Supplement: Supplementary file 1 [file am5c20819_si_001.pdf]

Unveiling the challenge of evaporator design in clean water production promoted by superabsorbent hydrogels and sunlight

Umamah Amir,[a,b] Sonia Lanzalaco,[a,b,] Kathrin Harre,[c] Alba Àgueda,[d] Maria M. Pérez-Madrigal,[a,b] Ignasi Sirés,[e] Elaine Armelin [a,b,*]*

[a] IMEM-BRT Group, Departament d'Enginyeria Química, EEBE, Universitat Politècnica de Catalunya (UPC), C/ Eduard Maristany, 10-14, Ed. I, 2nd floor, 08019, Barcelona, Spain.

[b] Barcelona Research Center in Multiscale Science and Engineering (CCEM), EEBE, Universitat Politècnica de Catalunya (UPC), C/ Eduard Maristany, 10-14, Basement S-1, 08019, Barcelona, Spain.

[c] Polymer Chemistry Laboratory, Faculty of Agriculture and Environment Chemistry, Hochschule für Technik und Wirtschaft Dresden (HTWD), Friedrich-List-Platz 1, D-01069, Dresden, Germany.

[d] CERTEC Group, Departament d'Enginyeria Química, EEBE, Universitat Politècnica de Catalunya (UPC), C/ Eduard Maristany, 10-14, Ed. I, 5th floor, 08019, Barcelona, Spain.

[e] Laboratori d'Electroquímica dels Materials i del Medi Ambient, Departament de Ciència de Materials i Química Física, Secció de Química Física, Facultat de Química, Universitat de Barcelona, Martí i Franquès 1-11, 08028 Barcelona, Spain.

*Corresponding authors: elaine.armelin@upc.edu and sonia.lanzalaco@upc.edu

2. EXPERIMENTAL SECTION

2.3. Physicochemical characterization

The equilibrium swelling ratio (ESR) of PNIPAAm-PAAm and PNIPAAm-PAAm/PEDOT:PSS hydrogels, placed inside a glass vial with tap, was determined by immersing the samples in 10 mL of Milli-Q water at a specified temperature for 24 h. The temperature of the container was controlled with a cryothermostat Julabo (F25-MC). After the swelling period, excess of surface water was carefully removed from the soft material, and the swollen samples were weighed in an analytical balance (precision 0.1 mg) to obtain the wet weight. To determine the dry weight, the previously swollen hydrogels were freeze-dried. Once completely dried, the samples were reweighed (equation S1) [26]

$$\boldsymbol{ESR}\ (\%) = \frac{\boldsymbol{W_s - W_d}}{\boldsymbol{W_d}} \times \mathbf{100} \qquad \textbf{(S1)}$$

where W_s and W_d are the weights of swollen and dry gel, respectively.

Fourier transform infrared (FTIR) spectroscopy was carried out with a Jasco 4700 spectrophotometer to observe the main absorption bands of the hybrid hydrogels. An attenuated total reflection accessory with a diamond crystal (Specac model MKII Golden Gate Heated Single Reflection Diamond ATR) was used to place the samples. The absorption of the lyophilised samples was measured in a wavenumber range from 4000 to 600 cm^{-1}, after 64 accumulation scans at 8 cm^{-1} of resolution, and after baseline correction.

Raman spectra were recorded using a Renishaw dispersive Raman microscope spectrometer (InVia Qontor, GmbH, Germany) operated via Renishaw WiRE software. The spectrometer is fitted with a Leica DM2700M optical microscope, a thermo-electrically cooled charge-coupled device (CCD) detector. A spectrograph dispersed the scattered light using diffraction gratings of 2400 lines mm^{-1} or 1200 lines mm^{-1}. Both dry and wet solar absorber hydrogel (SAHs) were analyzed using Raman spectroscopy. Both dry and wet SAHs were analyzed using Raman spectroscopy. The spectral range was set to 600–4000 cm^{-1} for dry samples and 2800–3800 cm^{-1} for wet samples to investigate hydrogen bonding interactions within the gel.

The internal morphology of the hydrogels was examined by scanning electron microscopy (SEM) was using a Zeiss Neon 40 focused ion beam (FIB) instrument equipped with an energy dispersive X-ray (EDX) system and operated at 5 kV. Freeze-dried hydrogel samples were cryo-fractured to obtain a cross-section of the samples, which were then mounted on the tip of the SEM using a double-side adhesive carbon disc. To attach the sample to the tip, silver paint was applied to one side of each sample, and then a thin layer of carbon was applied to prevent sample charging issues. To obtain pore size and distribution data, the images were processed using ImageJ software.

The absorbance curves of different ratio of solar absorber hydrogel (SAH) were obtained using a Shimadzu UV-3600 UV–vis–NIR spectrophotometer, equipped with an integrating sphere. Light absorbance was measured across a wavelength range of 250 to 2500 nm. Vacuum-dried hydrogel samples were used for the measurements, and the spectra were expressed in absorbance (%) versus wavelength (nm).

The porosity of hydrogels was determined by micro-computed tomography (micro-CT) analysis using a Skyscan 1272 (Bruker microCT) instrument. During the measurement, hydrogel samples were positioned horizontally, securely on the device table of the chamber. The measurement was conducted without the use of a filter at a 50 kV voltage, 10 W power, and 200 μA current. The step size of angle rotation was 0.2° for a single full turn to 180°, resulting in a voxel size of 3.00 μm in all samples and a frame averaging of 3. The cross-sectional images obtained from the micro-CT scan were reconstructed from projection images using NRecon software. Both 2D and 3D visualizations were generated with DataViewer and CTVox softwares, respectively. The structural analysis of the scaffolds was performed using CTAn software. All image analysis software employed in this study was sourced from Bruker micro-CT (Belgium).

Compression tests were conducted using a Shimadzu EZ-LX Tensiometer. Cyclic compression was applied to pre-prepared hydrogel samples at a rate of 1 mm min^{-1}. Cylindrical hydrogel samples (22 mm diameter, 10 mm height) were mounted between the compression plates of the testing machine, ensuring parallel alignment and uniform contact. Five cycles of loading and unloading for each sample were recorded, with a maximum strain of 50%. Force-displacement data were continuously recorded once the force reached 0.02 N. The hysteresis for each cycle was calculated by determining the area enclosed between the loading and unloading curves. The hysteresis loop area

represents the dissipation energy per unit volume upon deformation. Hysteresis (H, kJ/m^3) was calculated as the difference between the area under the loading curve and the area under the unloading curve for each stress–strain cycle. The hysteresis loss (HL, %) was obtained using the formula:

$$\boldsymbol{HL}\ (\%) = \frac{\boldsymbol{H}}{\boldsymbol{Area_{loading}}} \times \mathbf{100} \qquad \textbf{(S2)}$$

To evaluate fatigue behavior over five successive cycles, the change in hysteresis (ΔH) between the first (H_{5th}) and fifth (H_{1st}) cycles was calculated as:

$$\boldsymbol{\Delta H}\ (\%) = \frac{\boldsymbol{H_{5th} - H_{1st}}}{\boldsymbol{H_{1st}}} \times \mathbf{100} \qquad \textbf{(S3)}$$

The ΔH value indicates the extent of energy dissipation reduction across repeated loading, reflecting the hydrogel's fatigue resistance under cyclic mechanical stress [37]

Additionally, a uniaxial compression test was also conducted on the cylindrical hydrogels (same dimensions than previous, number of samples n = 4) at a compression rate of 1 mm min^{-1}. Testing continued until a strain of 70 % was reached and force – displacement data were recorded continuously after a force of 0.02N was reached. The elastic modulus and compressive strength were determined from the stress-strain curve in the elastic regime. All the samples were recovered without breaking.

A control experiment was conducted to estimate the vaporization enthalpy under dark conditions. Milli-Q water and PNIPAAm-PAAm (4:1)/PEDOT:PSS hydrogel with same surface areas were placed inside a chamber containing a supersaturated potassium carbonate solution to maintain a stable relative humidity of about 45% at room temperature (~22 °C). The equivalent evaporation enthalpy (E_{equ}) of water within the hydrogels was calculated by comparing their evaporation rates, using the known theoretical value of vaporization enthalpy of liquid water (E_0 = 2450 kJ kg^{-1}) and the equation below:

$$\boldsymbol{E_{equ}} = \boldsymbol{E_0} \frac{\Delta \boldsymbol{m_0}}{\Delta \boldsymbol{m_{hyd}}} \qquad \textbf{(S4)}$$

where E_0 is the equivalent evaporation enthalpy of bulk water, Δm_0 and Δm_{hyd} denote the mass change of water and PNIPAAm-PAAm (4:1)/PEDOT:PSS hydrogel, respectively.

The calculated E_{equ} was then used to evaluate the solar-to-vapor conversion efficiency. The solar thermal efficiency (η) was determined using:

$$\eta = \frac{\dot{m}(E_{equ} + C_p \quad \Delta T)}{q} \qquad \textbf{(S5)}$$

where $\dot{m}$ is the steady-state evaporation rate (kg m^{-2} s^{-1}), C_p is the specific heat capacity of water (4.20 × 10^3 J kg^{-1} K^{-1}), ΔT is the temperature rise, and q represents the solar energy (1 sun = 1 kW m^{-2}). Therefore, as our system does not follow a constant temperature equilibrium over SDD with time (due to the hydrogel top surface shrinkage with T>T_{LCST}), the η was calculated with time (here called "dynamic efficiency"). The results obtained for ER_{2D} and ER_{3D} hydrogel area with time and how it affects the thermal efficiencies were discussed in section 3.4 (main text).

3. RESULTS and DISCUSSION

3.1. Preparation and characterization of superabsorbent hydrogels

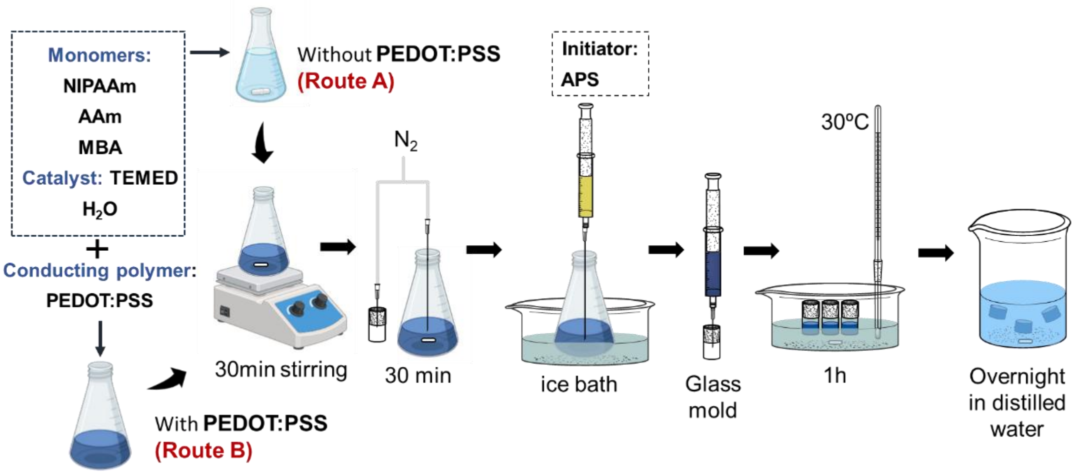

Figure S1. Schematic representation of the procedure carried out to prepare the PNIPAAm-PAAm, without (Route A) and with (Route B) conducting polymer.

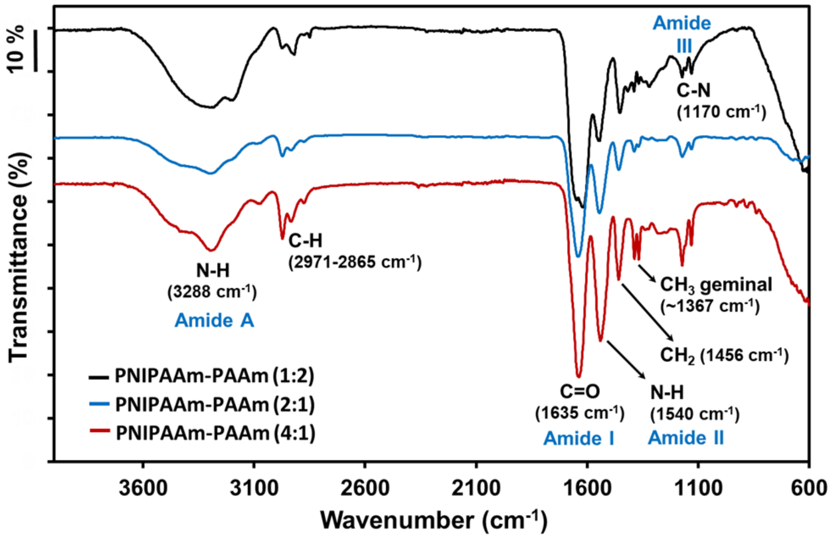

Figure S2. FTIR spectra of PNIPAAm-PAAm copolymer, with different compositions and without conducting polymer.

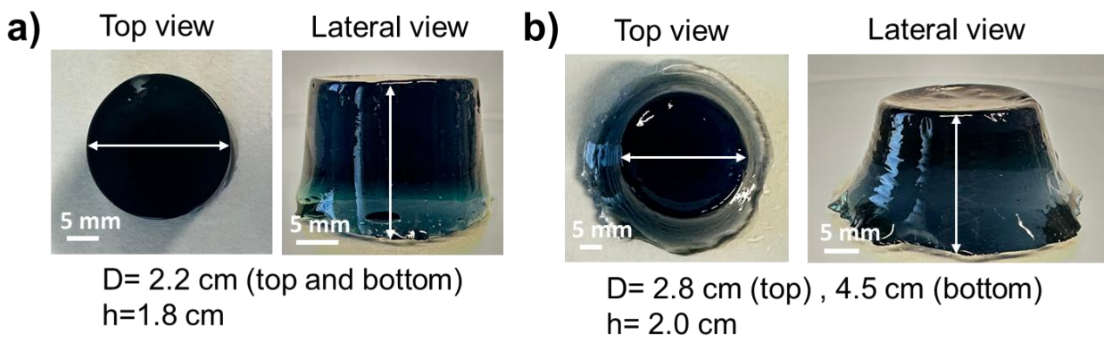

Figure S3. Photographs of PNIPAAm-PAAm (2:1)/PEDOT:PSS hydrogel: a) before and b) after 24 h of immersion in seawater, proving their great capacity of seawater uptake (superabsorbent property).

3.3. UV absorption and the mechanical deformation of superabsorbent, thermoresponsive and solar absorber PNIPAAm-PAAm/PEDOT:PSS hydrogels

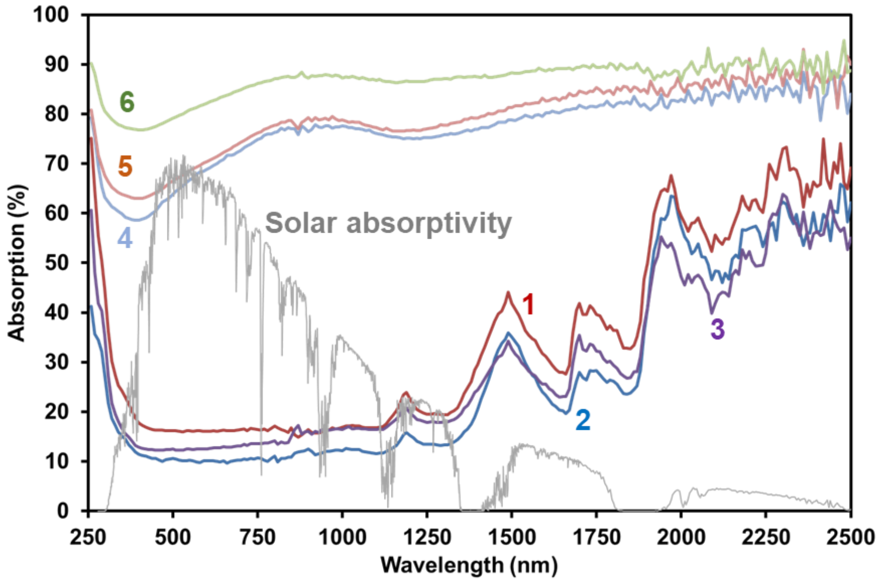

Figure S4. UV-visible spectra of all samples and solar absorptivity in the range of 250 nm to 2500 nm. Codes: 1) PNIPAAm-PAAm (1:2); 2) PNIPAAm-PAAm (2:1); 3) PNIPAAm-PAAm (4:1); 4) PNIPAAm-PAAm (1:2)/PEDOT:PSS; 5) PNIPAAm-PAAm (2:1)/ PEDOT:PSS; and 6) PNIPAAm-PAAm (4:1)/PEDOT:PSS.

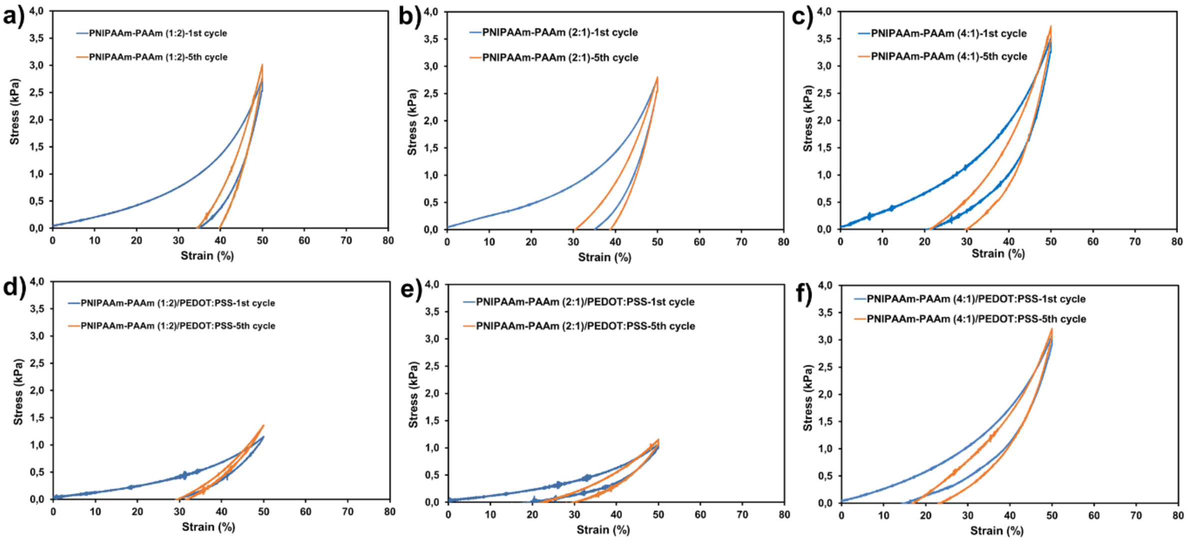

Figure S5. Cyclic compression curves (1st and 5th cycle) for: a) PNIPAAm-PAAm (1:2); b) PNIPAAm-PAAm (2:1); c) PNIPAAm-PAAm (4:1); d) PNIPAAm-PAAm (1:2)/PEDOT:PSS; e) PNIPAAm-PAAm (2:1)/ PEDOT:PSS; and f) PNIPAAm-PAAm (4:1)/PEDOT:PSS.

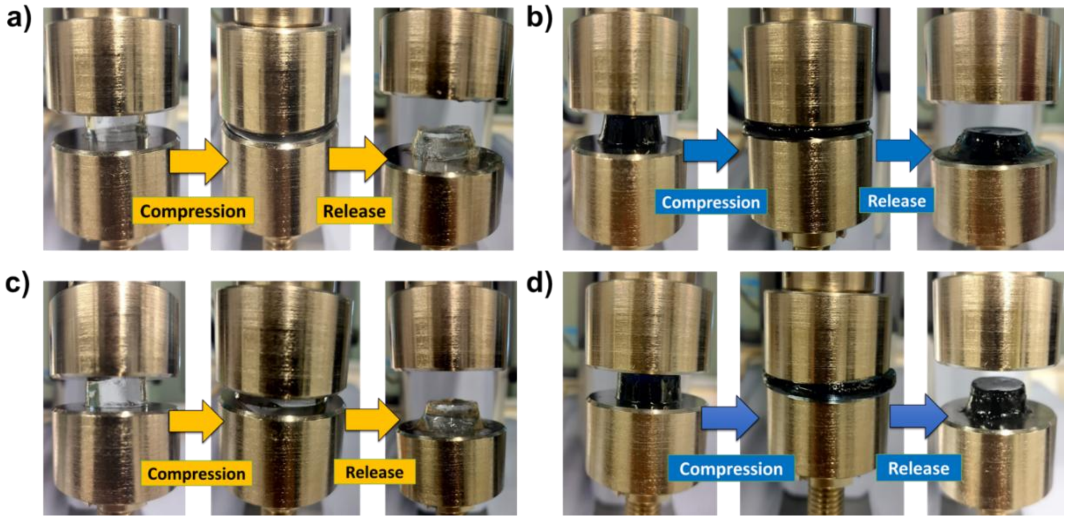

Figure S6. Photographs of the compression and release during the 1st cycle in compression testing: a) PNIPAAm-PAAm (1:2); b) PNIPAAm-PAAm (2:1); c) PNIPAAm-PAAm (1:2)/PEDOT:PSS; and d) PNIPAAm-PAAm (2:1)/ PEDOT:PSS hydrogels.

Table S1. Mechanical properties of superabsorbent hydrogels under cyclic compression tests.

PNIPAAm-PAAm					
Molar ratio	Young modulus (kPa)	Strength (kPa)	Hysteresis 1st / 5th cycle (kJ/m^3)	HL 1st / 5th cycle (%)	ΔH (%)
1:2	1.47 ± 0.14	10.15 ± 0.92	26.6 / 6.9	67.1 / 37.6	-73.9
2:1	1.65 ± 0.16	11.75 ± 0.90	29.0 / 9.4	68.5 / 45.5	-67.3
4:1	2.34 ± 0.24	9.87 ± 1.83	30.7 / 15.5	53.7 / 40.9	-49.4
PNIPAAm-PAAm/PEDOT:PSS					
Molar ratio	Young modulus (kPa)	Strength (kPa)	Hysteresis 1st / 5th cycle (kJ/m^3)	HL 1st / 5th cycle (%)	ΔH (%)
1:2	1.21 ± 0.06	7.60 ± 1.00	11.6 / 1.6	57.2 / 13.8	-86.3
2:1	1.26 ± 0.11	6.80 ± 0.5	9.5 / 4.8	50.5 / 38.2	-49.6
4:1	2.48 ± 0.38	10.96 ± 2.52	21.2 / 13.8	42.0 / 34.6	-35.1

3.4. Solar-driven evaporation in open air comparing three models of SVG arrangements

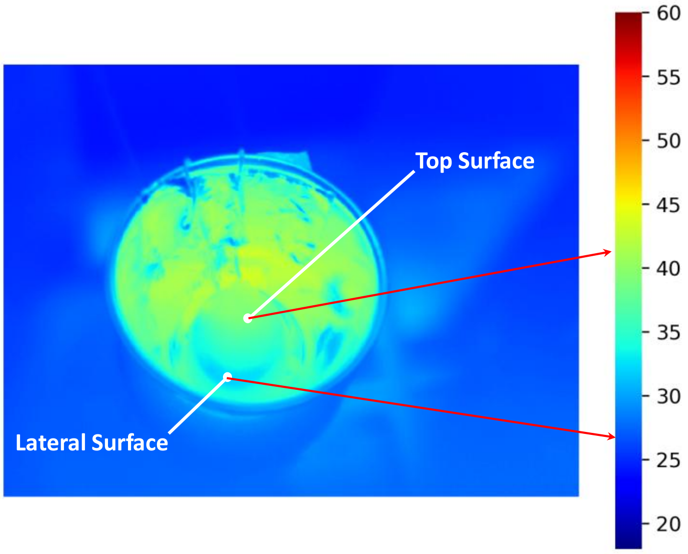

Figure S7. Infrared thermal image of PNIPAAm-PAAm (4:1)/PEDOT:PSS hydrogel employed using the assembly Model II.

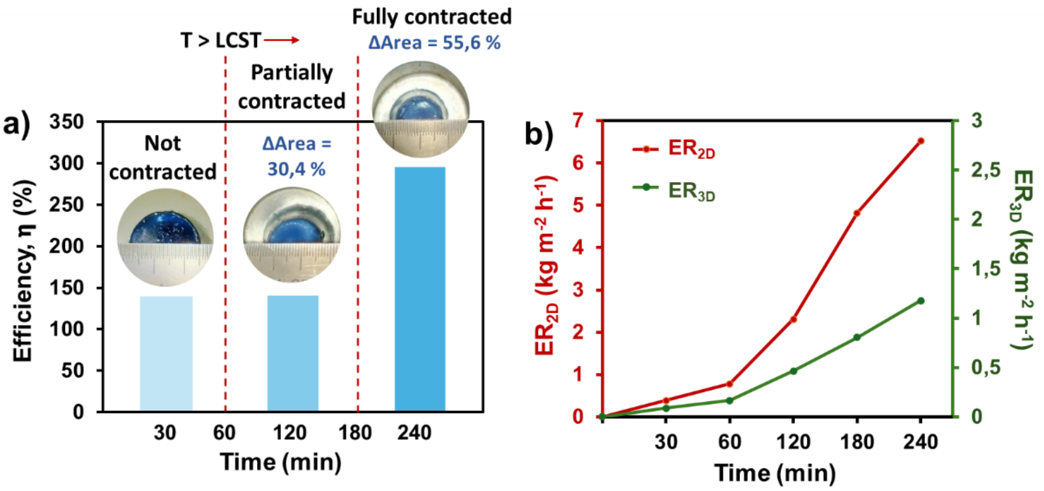

Figure S8. A) Dynamic efficiency (η) data, calculated with time for PNIPAAm-PAAm (4:1)/PEDOT:PSS hydrogel top surface (ER_{2D}) under solar irradiation. b) Increasing ER_{2D} and ER_{3D} profiles of PNIPAAm-PAAm (4:1)/PEDOT:PSS hydrogel over the time of sunlight irradiation.
